# Diacylglycerols interact with the L2 lipidation site in TRPC3 to induce a sensitized channel state

**DOI:** 10.15252/embr.202154276

**Published:** 2022-05-23

**Authors:** Hazel Erkan‐Candag, Amy Clarke, Oleksandra Tiapko, Mathias AF Gsell, Thomas Stockner, Klaus Groschner

**Affiliations:** ^1^ Gottfried‐Schatz‐Research‐Center – Biophysics Medical University of Graz Graz Austria; ^2^ Institute of Pharmacology Medical University of Vienna Vienna Austria

**Keywords:** diacylglycerol, lipid regulation, lipid–protein interactions, TRPC channels, Membranes & Trafficking, Signal Transduction

## Abstract

Coordination of lipids within transient receptor potential canonical channels (TRPCs) is essential for their Ca^2+^ signaling function. Single particle cryo‐EM studies identified two lipid interaction sites, designated L1 and L2, which are proposed to accommodate diacylglycerols (DAGs). To explore the role of L1 and L2 in TRPC3 function, we combined structure‐guided mutagenesis and electrophysiological recording with molecular dynamics (MD) simulations. MD simulations indicate rapid DAG accumulation within both L1 and L2 upon its availability within the plasma membrane. Electrophysiological experiments using a photoswitchable DAG‐probe reveal potentiation of TRPC3 currents during repetitive activation by DAG. Importantly, initial DAG exposure generates a subsequently sensitized channel state that is associated with significantly faster activation kinetics. TRPC3 sensitization is specifically promoted by mutations within L2, with G652A exhibiting sensitization at very low levels of active DAG. We demonstrate the ability of TRPC3 to adopt a closed state conformation that features partial lipidation of L2 sites by DAG and enables fast activation of the channel by the phospholipase C‐DAG pathway.

## Graphical abstract

Diacylglycerols (DAGs) sensitize TRPC channels by interactions with the L2 lipidation site in the channel´s pore domain. The DAG sensitized state enables fast channel activation and a high‐activity Ca^2+^ signaling mode.

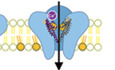

## Introduction

The crucial role of lipids protruding from the bilayer into cavities and fenestrations of ion channel transmembrane domains is becoming increasingly recognized. Such “structural” or “non‐annular” lipids, which interact with key structures of ion channels including gating elements and parts of the permeation pathway, have been detected by high‐resolution structure analysis for various ion channel complexes. These lipid–protein interactions stabilize important functional states of ion channels and have been identified as determinants of regulatory and pharmacological features (Poveda *et al*, 2014; Svobodova & Groschner, 2016; Lichtenegger *et al*, 2018). An outstanding example of the concept of coordination of regulatory lipids within transmembrane elements of ion channels are members of the transient receptor potential canonical (TRPCs) family of Ca^2+^‐permeable cation channels (Storch *et al*, 2017; Mederos y Schnitzler *et al*, 2018). For the isoforms TRPC2/3/6/7, the lipid mediator diacylglycerol (DAG) has been recognized as a prominent and most likely direct channel activator. DAG‐channel interactions are proposed to exert primary control over channel gating (Hofmann *et al*, 1999; Wang *et al*, 2020). Thus, TRPC2/3/6/7 have been classified as “second‐messenger operated channels” and may, more precisely, be designated as “primarily lipid‐gated Ca^2+^ channels”. Interestingly, DAG‐induced activation has also been observed for TRPC4 and TRPC5, but only when this regulatory feature is uncovered by uncoupling these channel complexes from interacting NHERF proteins (Storch *et al*, 2017). Deciphering the unique principle of direct lipid‐gating of channel proteins is essential for an in‐depth understanding of TRPC Ca^2+^ signaling, but more generally for comprehending the role of these channels in health and disease.

**Figure 1:**
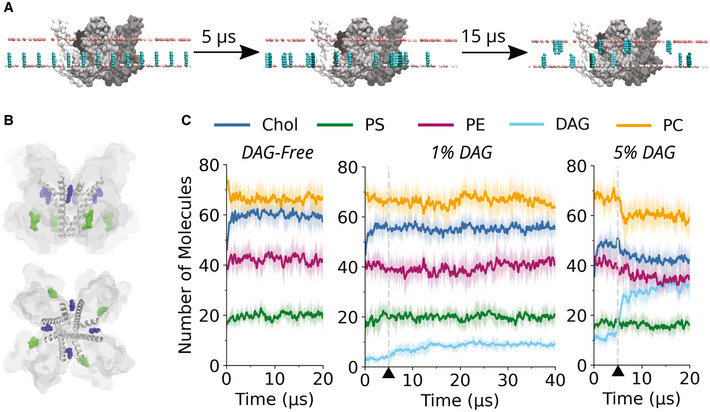
MD simulations provide evidence for DAG‐TRPC3 interactions Strategy of the MD simulations. Coarse‐grained TRPC3 (tetramer, shown in gray) is embedded in membranes with either 10% or 2% DAG (blue) in the inner leaflet (equivalent to 5% and 1% of total membrane lipids, respectively) (Appendix Table S2). Position restraints applied only on the Z‐axis keep the DAG in the inner leaflet for the first 5 µs of the simulation. After this, the position restraints are removed and DAG is able to flip into the outer leaflet, and the simulations are continued for either a further 15 µs or 35 µs. Light pink beads represent the phosphate groups of the phospholipids.Image of TRPC3 in surface representation showing the two lipid binding sites as identified in the cryo‐EM structure with the PDB ID: 6cud. Shown in gray are the pore and S6 helices. Two lipid entities are highlighted in green and blue, these represent the lipid densities at the L1 (green) and L2 (blue) binding sites identified in the cryo‐EM structure.The average number of lipid molecules within 0.6 nm of the TRPC3 tetramer as a function of time, for the DAG‐free, 5% DAG, and 1% DAG membranes. In dark is the mean averaged across five repeats, with a window average of 200 ns; in light is the standard deviation. Triangles and dashed gray lines indicate the 5 µs time point when the position restraints are removed from DAG. The abbreviations used in this figure are: PC (phosphatidylcholine), Chol (cholesterol), PE (phosphatidylethanolamine), PS (phosphatidylserine), and DAG (diacylglycerol, specifically stearoyl‐arachidonoyl glycerol).

Despite the early proposal of a direct mechanism of DAG‐induced channel activation (Hofmann *et al*, 1999), the process of DAG sensing for these channels has remained largely enigmatic over the past decades. Besides speculations about cytosolic DAG sensing structures (Svobodova & Groschner, 2016), indirect mechanisms involving lipid‐induced changes in membrane structure and mechanics have also been suggested (Spassova *et al*, 2006). The first evidence for a DAG‐sensing structure residing within the transmembrane region of TRPC3 channels came from a functional study using lipid photopharmacology (Lichtenegger *et al*, 2018) and, in parallel, from TRPC3 structure analysis by single particle cryo‐EM (Fan *et al*, 2018). Both approaches localized critical lipid interactions within a lateral fenestration of the TRPC3 pore domain (Groschner & Tiapko, 2018). Nonetheless, current structural models of TRPC3 and TRPC6 tetramers feature two distinct lipid coordination sites termed L1 and L2, which reside within the voltage sensor‐like domain and the pore domain of the channel, respectively (Fig 1B). However, as yet the lipid species coordinated within these sites have not been clearly identified and the ability of DAG to govern TRPC3 function by protein–lipid interactions in one or both of these sites remains to be demonstrated.

Here, we set out to test this concept by combining molecular dynamics (MD) simulations and simulation‐guided mutagenesis with photopharmacological analysis of DAG‐induced activation kinetics. We provide the first evidence for rapid accumulation of DAG within transmembrane domains of TRPC3, most prominently at L2, once the lipid mediator becomes available within the inner leaflet surrounding the channel complex. Moreover, we resolved two components of DAG‐induced channel activation, including a thus far unrecognized sensitizing process, which involves L2 lipidation and prepares TRPC3 channels for fast activation and efficient Ca^2+^ signaling.

## Results and Discussion

### Molecular dynamics simulations of the interactions of TRPC3 with membrane lipids

In order to understand the behavior of the lipid mediator DAG in membranes and probe possible interaction sites with the primarily DAG‐gated TRPC3, we undertook extensive coarse‐grained (CG) MD simulations. We generated three sets of complex, asymmetric membranes of five repeats each (Appendix Table S2). Regulatory DAG is generated *in vivo* in the inner leaflet by phospholipase C (PLC)‐mediated cleavage of phosphatidylinositol‐4,5‐bisphosphate (PIP_2_), which is present in the inner leaflet at a concentration of 2% (or 1% of total membrane lipids). To reflect this, DAG, specifically stearoyl‐arachidonoyl glycerol (SAG), was placed at random positions in the inner leaflet at the start of the simulations, at concentrations of 10% and 2% of the inner leaflet lipids (corresponding to 5% and 1% of total lipid content, respectively), which is correlated with the concentration of the parent lipid PIP_2_ (approximately 2% in the inner leaflet) and in line with lipidomic studies of mammalian cells (Sampaio *et al*, 2011). As DAG can flip between leaflets of the membrane without the requirement for a lipid transporter, a phenomenon that is captured in CG MD, position restraints along the Z‐axis were used to confine DAG to the inner leaflet during the first 5 µs of the simulations, to allow phospholipids and cholesterol to equilibrate in the presence of TRPC3 (Fig 1A). After 5 µs, the position restraints were removed from DAG, and DAG was able to diffuse freely between the inner and outer leaflets. The simulations were then continued for a further 15 µs for the DAG‐free and 5% DAG membranes, and a further 35 µs for the 1% DAG membranes.

### Spatiotemporal aspects of lipid‐TRPC3 contact formation

The number of lipid molecules within 0.6 nm of TRPC3 as a function of time was analyzed (Fig 1C). The analysis serves a two‐fold purpose: firstly, it allows us to determine when the lipid molecules and TRPC3 have reached an equilibrium; secondly, it gives a general picture of which lipid species are enriched in the inner shell and within the hydrophobic cavities and clefts of the protein. From this analysis, it is clear that for the DAG‐free, four‐component membrane (Appendix Table S2), the lipids and TRPC3 have reached an equilibrium after 5 µs. In the case of the 1% and 5% DAG containing membranes, an equilibrium is reached after approximately 15 µs. Moreover, these data suggest that DAG binds rapidly to TRPC3 upon its availability to interact, after removal of position restraints, even at a concentration as low as 1% of total membrane lipids. This may reflect specific molecular features of the lipid mediator, most prominently its hydrophobicity, which not only allows it to flip in the membrane without the requirement of a lipid transporter but also to protrude into hydrophobic grooves of TRPC3.

**Figure 2:**
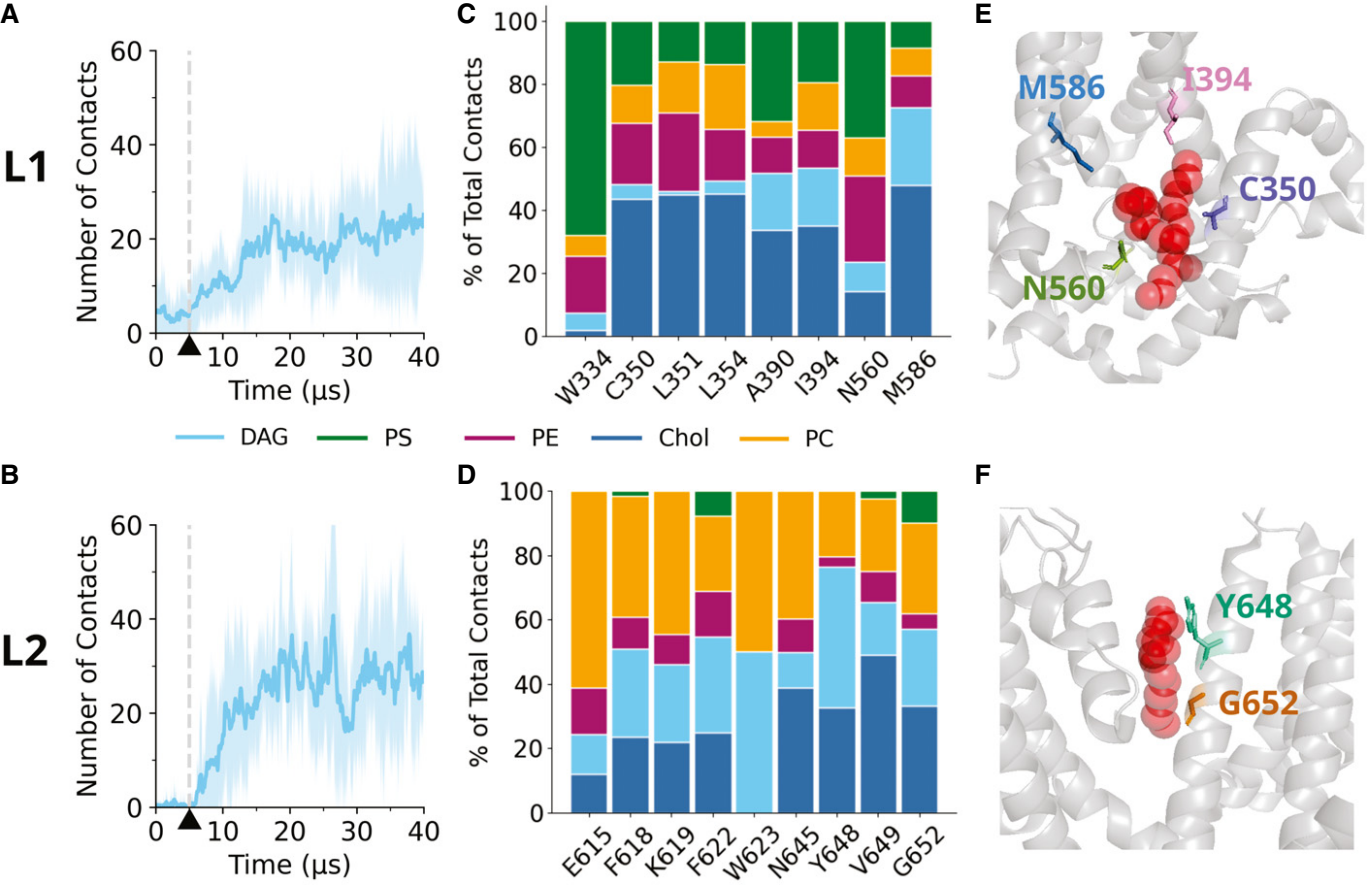
DAG interacts with residues within the L1 and L2 coordination sites AAverage number of contacts between DAG and the L1 residues as a function of time in a membrane containing 1% DAG. A cutoff of 0.6 nm has been used to define a contact. The L1 residues refer to W334, C350, L351, L354, A390, I394, N560, and M586. Dark blue shows the mean averaged from five repeats; light blue represents the standard deviation. A window average of 200 ns has been applied. Dashed gray line and black triangle indicates the 5 µs time point when the position restraints are removed from DAG.BAs in (A), except for the L2 residues, which refer to E615, F618, K619, F622, W623, N645, Y648, A649 and G652. Dashed gray line and black triangle indicates the 5 µs time point when the position restraints are removed from DAG.C, DThe relative contribution of each lipid species to contacts at the identified residues of the L1 (C) and L2 (D) binding site.E, FResidues identified as candidates for mutagenesis for (E) the L1 and (F) L2 binding sites, respectively. In red are the lipid densities identified in the cryo‐EM structure (Fan *et al*, 2018).

**Figure EV1:**
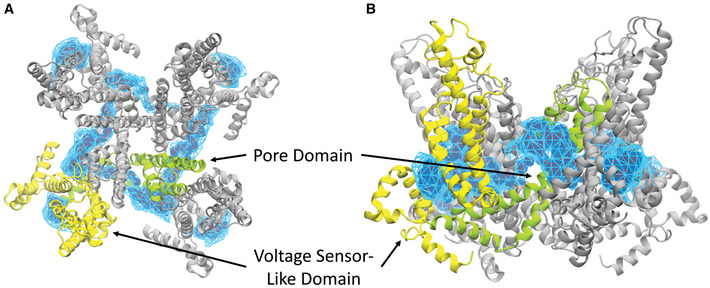
Three‐dimensional density maps showing DAG interaction with TRPC3 A, BTRPC3 is shown in gray, with one subunit highlighted in yellow (voltage sensor‐like domain) and green (pore domain). Blue represents DAG density. (A) Top view (B) Side view.

Next, we focused specifically on the L1 and L2 lipid binding sites, recently identified in cryo‐EM studies (Fan *et al*, 2018). From the available structural information, we considered eight residues of the L1 and nine residues of the L2 as potentially involved in coordination of non‐annular lipids. We calculated the number of contacts between DAG and the L1 or L2 binding sites as a function of time; a contact is considered when two coarse‐grained beads come within 0.6 nm of each other. Our analysis suggests that at a concentration of 1%, DAG binds to both the L1 and L2 binding sites (Fig 2A and B), which was further confirmed by the calculation of three‐dimensional DAG density profiles (Fig EV1A and B). We also considered contacts between these residues and the phospholipids and cholesterols in the membrane. Taking the final 25 µs of the simulation (after the lipids and TRPC3 have reached an equilibrium), we calculated the total number of contacts that each residue establishes. We then calculated the percentage of these contacts accounting for DAG interactions, and the percentage comprised of phospholipid and cholesterol interactions (Fig 2C and D). From this, it becomes clear that, despite only being present at concentration of 1% of total membrane lipids, DAG can contribute significantly to interactions at the L2 binding site (Dietrich *et al*, 2003; Rosker *et al*, 2004).

### Identification of amino acid residues potentially involved in DAG‐TRPC3 interaction

The coarse‐grained MD simulations provided evidence for the ability of DAG to accommodate rapidly within the L1 and L2 binding sites of TRPC3 once the lipid mediator becomes available as an interaction partner. Importantly, DAG‐TRPC3 contact formation was found to be more prominent in the L2 as compared to the L1 (Fig 2C and D). Interestingly, in addition to DAG, we also observed accommodation of other lipids within L1 and L2, particularly cholesterol (Chol), phosphatidylcholine (PC), and phosphatidylserine (PS), as illustrated in Fig 2C and D. Hence, it appears plausible that, when DAG unbinds from coordination sites, another most likely functionally inert lipid occupies its space. In order to explore the functional role of the predicted occupancy of L1 or L2 sites by DAG, we set out to compare the function of wild‐type TRPC3 with that of channels carrying single point mutations predicted to interfere with DAG coordination. Applying an MD‐guided mutagenesis strategy, we exchanged amino acid residues in positions that were suggested as DAG contact sites by the simulations. We selected four residues within L1 (C350, I394, N560, and M586), which were predicted to account for a major fraction of DAG contacts (Fig 2C and E). For L2, we chose two residues, based on available information on the sensitivity of this region to mutagenesis (Fig 2F). As a complicating factor, the L2 coordination site resides in a critical, conserved region of the TRPC3 pore domain including a highly sensitive LFW motif, which most likely contains DAG binding residues (L622 and F623). Exchange of these critical residues in the L2 domain is reported to eliminate membrane targeting or channel function. Nonetheless, we were able to identify two DAG binding‐competent L2 residues (Y648 and G652), which were suitable for structure‐function analysis. Our previous mutagenesis analysis within the TRPC3 pore domain revealed substantial alterations in the DAG recognition features for the G652A mutation (Lichtenegger *et al*, 2018), which was found to suppress activation by endogenous DAGs while facilitating activation by certain exogenous DAG molecules including photochromic ligands. MD simulations revealed a significant fraction of DAG interactions for G652, which was previously suggested as part of a DAG sensor in TRPC3 (Hofmann *et al*, 2002; Strübing *et al*, 2003). In addition to G652, we selected Y648, which was identified by MD simulations as a residue that stands out for its particularly high propensity to form contacts with DAG. Figure 2E and F illustrates the localization of the amino acids chosen for mutagenesis.

### Mutations within L1 and L2 generate a loss of TRPC3 channel function

**Figure 3:**
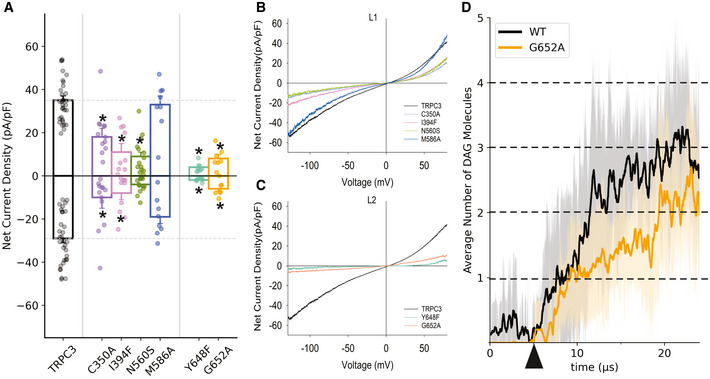
Mutations in both lipid coordination sites (L1 and L2) impair TRPC3 channel function AMean net current densities of CCh (200 μM)‐stimulated inward and outward currents through TRPC3‐WT (black) and mutant channels (color‐coded as indicated) expressed in HEK293 cells. Values from individual experiments are shown for each of the columns (circles). Number of biological repetitions ≥ 6. Data are mean ± SEM; two‐tailed *t*‐test or Mann–Whitney test were applied between TRPC3‐WT and mutants; *P < 0.05.B, CRepresentative I–V relations of carbachol (CCh)‐induced net current densities (200 μM) through L1 mutants (B) and L2 mutants (C) expressed in HEK293 cells. TRPC3 WT is shown in black. The insert shows the mean reversal potential of illustrated currents.DAverage number of DAG molecules within 0.6 nm of the L2 binding sites of the TRPC3 tetramer as a function of time for a membrane containing 1% DAG. Release of DAG positional restraint in the inner leaflet is marked by triangle and number of DAG molecules in the tetramer (level of lipidation) is indicated by dashed lines. In dark is the mean averaged across five repeats, with a window average of 200 ns; in light is the standard deviation.

To replace residues predicted to form contacts with DAG in the L1/L2, we selected amino acid types based on structural considerations aimed at (i) preserving protein folding by considering secondary structure propensity and (ii) maintaining interference with lipid coordination by taking side chain polarity/hydrophobicity into account. These TRPC3 mutants were first tested for their ability to retain wild‐type like membrane targeting. First, we analyzed membrane localization of N‐terminally fused YFP variants expressed in HEK293 cells using epifluorescence and TIRF imaging. All generated mutant channels displayed clear plasma membrane targeting as illustrated in Appendix Fig S1A. Consistently, all TRPC3 mutants displayed significant albeit variable ability to generate membrane conductances when activated by the synthetic agonist GSK1702934A, thereby demonstrating that the channel function remained preserved (Appendix Fig S1B). Next, we set out to characterize these mutant channels in the context of their response to activation by endogenous DAGs produced via PLC activation through stimulation with the muscarinic receptor agonist carbachol (CCh, 200 μM). The conductance of TRPC3 mutants showed significantly reduced peak current densities compared to wild‐type (Fig 3). Except for one L1 mutation (M586A) all mutants displayed reduced inward and outward currents, with the I‐V characteristics showing a barely altered shape. L2 mutants displayed profoundly reduced activity as compared to wild‐type TRPC3 (Fig 3A–C). In order to correlate the L2 G652A mutant phenotype with molecular details of DAG‐TRPC3 interactions, we used MD simulations and compared the DAG interactions in wild type and G652A channels. As illustrated in Fig 3D, the G652A mutation displayed clearly reduced overall occupancy by DAG (SAG) in the tetrameric complex, corresponding to the observed blunted activation by endogenous DAG in response to CCh stimulation in our electrophysiological experiments. It is important to note that this altered DAG interaction was revealed for the closed channel complex. Hence, our simulation‐guided mutagenesis experiments indicated a dominant role of L2 in DAG‐mediated regulation of TRPC3. Mutations within L2, which were predicted to compromise lipid coordination, exerted a more profound impact on PLC‐DAG mediated channel activation as compared to L1 mutations.

### Optical Lipid Clamp revealed a sensitization process induced by repetitive photostimulation in WT and mutants

Selected mutations in TRPC3, which are predicted to affect the lipidation of the channel in either L1 or L2, caused a loss of channel function. This could either reflect alterations in the lipidation pattern of the respective coordination site, including impaired DAG binding, or it could reflect an allosteric, structural distortion of the lipid gating machinery of TRPC3. Therefore, we set out to characterize the functional consequence of the L1 and L2 mutations in greater detail, using a recently established photo‐pharmacological approach designated “optical lipid clamp” (Lichtenegger *et al*, 2018). For a more sensitive and direct test that aims to reveal a role of L1 and/or L2 in DAG‐mediated channel activation, we studied the conductance activation of point mutations during repetitive photocycling of a recently developed photoswitchable DAG (OptoDArG). We hypothesized that genetic modification of a functionally relevant DAG coordination site will affect the kinetics of channel activation due to altered on‐rate for DAG accommodation within the regulatory site. OptoDArG was previously identified as a DAG species that generates particularly large and robust TRPC3 channel activation and displays enhanced activity in the G652A mutant (Lichtenegger *et al*, 2018). The reported tendency to generate larger current amplitudes makes OptoDArG a preferred tool for a detailed analysis of the kinetics of DAG‐induced channel activation.

**Figure 4:**
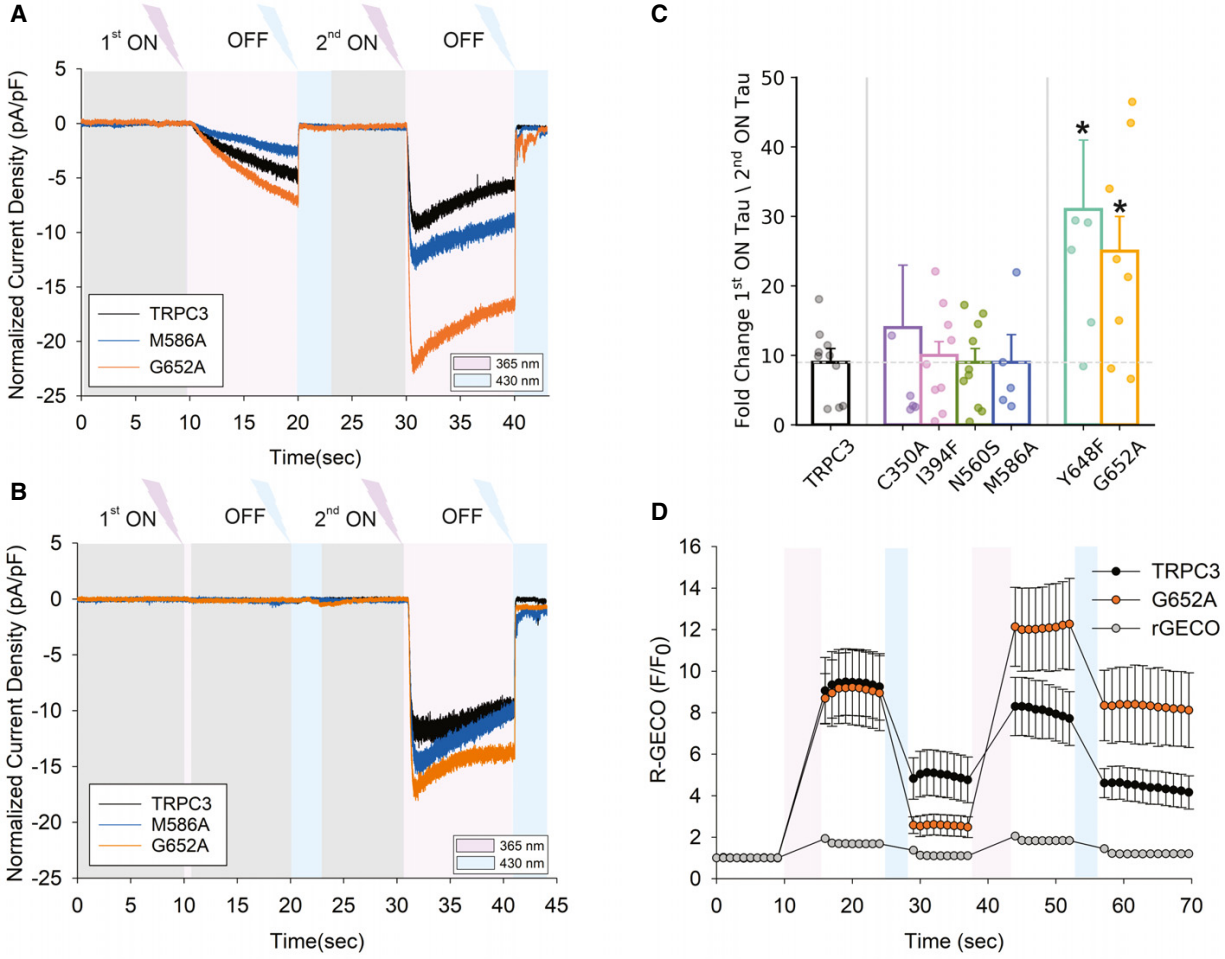
Optical lipid clamp unravels a sensitization process based on DAG‐TRPC3 interactions Representative recordings showing the inward currents induced by repetitive photoactivation of OptoDArG (20 μM) in a whole‐cell, gap‐free recording (holding potential: − 40 mV, normalized by capacitance) in TRPC3‐WT, M586A and G652A‐expressing HEK293 cells. 100% intensity of UV: 365 nm for 10 s (violet), blue light: 430 nm for 10 s (blue).Representative time courses of inward currents induced along with a reduced first photoactivation of OptoDArG. Initial photocycling involved illumination with 30% intensity of UV for 1 s (365 nm) followed by blue light (430 nm, 3 s, blue). Second photocycling was induced by 100% UV (365 nm) for 10 s and blue (430 nm) for 3 s.Columns illustrate the fold change in Tau ON between first and second current activation corresponding to enhanced speed of current activation for WT and mutant channels as indicated. Number of biological repetitions for each condition ≥ 6. Data are mean ± SEM; two‐tailed *t*‐test or Mann–Whitney test were applied; *P < 0.05.OptoDArG‐induced Ca^2+^ signals generated by repetitive activation of TRPC3 WT and G652A using R‐GECO as a Ca^2+^ reporter and the illumination protocol corresponding to (A). Effects in cells expressing R‐GECO only are shown as a control. Number of biological repetitions for each condition ≥ 6. Data are mean ± SEM.

**Figure EV2:**
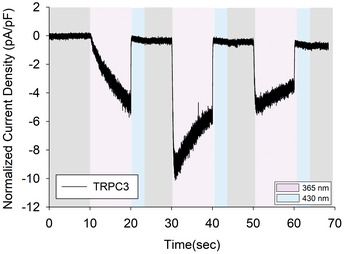
Time course of WT TRPC3 during 3 repetitive activating photocycles Representative recordings showing the inward currents induced by 3 repetitive photoactivation of OptoDArG (20 μM) in a whole‐cell, gap‐free recording (holding potential: − 40 mV, normalized by capacitance) in TRPC3‐WT‐expressing HEK293 cells. 100% intensity of UV: 365 nm for 10 s (violet), blue light: 430 nm for 10 s (blue).

The optical lipid clamp approach enables temporally precise exposure of membrane proteins to regulatory lipids. OptoDArG is a potent and efficient TRPC3 activator in its cis conformation, but inactive when switched to the trans conformation. Figure 4A illustrates the time courses of TRPC3 channel activation during repetitive OptoDArG photocycling. To ensure that channels lack any pre‐activation from traces of cis OptoDArG, we introduced a 20‐sec pre‐illumination step (blue light; Pre‐Blue, 430 nm) before the experiments to quantitatively convert all OptoDArG into the inactive trans conformation (not included in time course illustrations). Figure 4 depicts the time course of current activation during two consecutive activating pulses by UV light (10 s; 365 nm) to generate cis OptoDArG. Each activation period was terminated by returning OptoDArG to its inactive trans conformation by blue light illumination (430 nm) as indicated. We observed strikingly divergent activation characteristics for the first and second activation cycles, with a complex, slow activation kinetics for the initial photoactivation and a substantially faster activation associated with enhanced maximum current densities for the second activation cycle (Fig 4A). These results suggested a sensitization process that is triggered by the first exposure of the channels to DAG and the existence of a sensitized state, displaying fast activation. This channel state persisted during subsequent activating pulses (Fig EV2). The first activation (“on”) kinetics was best fitted by a power exponential function combined with a linear component, representing a rather slow process that overlaps with the exponential kinetics expected for classical ligand gating. The second “on” kinetics, proposedly representing the activating process of lipid gating, was sufficiently well fitted by a power exponential. The observed sensitization/potentiation phenomenon may be explained either by a mechanism that depends on channel opening (current dependent) or that depends on a DAG interaction with the TRPC3 channels. A sensitizing DAG‐TRPC3 interaction may take place in their closed conformation as our MD simulations provided clear evidence for DAG coordination within the closed TRPC3 complex. Hence, to further investigate the basis of the sensitization phenomenon, we reduced the intensity of the first activating pulse to a level below or close to the threshold of conductance activation (Fig 4B). A 1 s UV illumination pulse at 30% intensity generated this low level of cis OptoDArG close to the threshold that was clearly sufficient to enable a subsequent fast activation of the channels. We therefore concluded that the observed TRPC3 sensitization was due to DAG‐TRPC3 interaction. Importantly, evaluation of the degree of sensitization, determined as the ratio between the time constants for first and second channel activation, uncovered L2 as a determinant and potential interaction site responsible for channel sensitization. Mutations in L2 but not in L1 promoted DAG‐induced channel sensitization as shown in Fig 4C. Collectively, the prominent DAG interactions in L2 as revealed by the MD simulations and the profound impairment of PLC‐mediated channel activation by L2 mutations not only corroborated the previously proposed role of L2 for TRPC3 regulation, but in addition suggested L2 as a target site for an as yet unrecognized process of channel sensitization. The prominent role of L2 was also evident from a direct comparison of kinetic parameters deduced from the lipid clamp experiments. Both L2 mutations but none of the tested L1 mutations significantly affected “on” kinetics of 1st or 2nd channel activation (Appendix Table S1). Of note, the power of exponential fits for the first activation was close to 4 (3.6 for WT), while the second activation displayed higher variability with values ranging from 2 (WT) to 10 (Y648A). This may be interpreted as the requirement of conformational changes in all four channel subunits during the first activation process, whereas structural rearrangements within only two of the four channel subunits are required for the final opening of the sensitized channels.

To evaluate the consequences of DAG‐mediated TRPC3 channel sensitization for cellular Ca^2+^ signaling, we measured Ca^2+^ entry into HEK293 cells expressing WT TRPC3 and the L2 mutant G652A activated during repetitive activation by OptoDArG (Fig 4D). For the L2 mutant G652A, which displayed the most profound sensitization in terms of accelerated current activation and enhanced current amplitude, cellular Ca^2+^ rises were substantially enhanced during a second activation cycle. This suggests that the observed channel sensitization translates into altered Ca^2+^ signaling. In contrast, Ca^2+^ signal potentiation was not observed with TRPC3 WT channels, which displayed similarly accelerated current activation, but only minor potentiation of the maximum current amplitude (Fig 4A). It is important to note that the readout in the experiments was global cytoplasmic Ca^2+^, while for many TRPC signaling cascades local Ca^2+^ changes and specifically the temporal pattern of local Ca^2+^ is of critical importance. It appears therefore plausible to speculate that the observed modulation of TRPC3 kinetics, generated by DAG‐induced sensitization, is likely to affect all cellular functions of TRPC3 that rely on localized Ca^2+^ signal patterns such as Ca^2+^ transcription coupling (Poteser *et al*, 2011) or neuronal frequency modulation (Neuner *et al*, 2015; Tiapko & Groschner, 2021).

The above‐described results lead us to hypothesize that DAG, at low levels, is able to interact with the L2 lipid coordination site within TRPC3 and to induce a sensitized state without opening of the permeation pathway. At higher DAG levels, the lipid mediator presumably increases the open probability of TRPC3 by an additional DAG‐induced conformational change within the channel complex. The final DAG‐induced channel opening may require saturation of DAG coordination sites within the tetrameric complex. These processes apparently overlap during an initial, instantaneous exposure to DAG levels that are sufficient for channel opening, as represented by the first activation in the double‐pulse optical‐lipid clamp experiments. During a subsequent activating UV pulse (second activation) TRPC3 channels (wild type and mutants) open with fast kinetics (Appendix Table S1), ‐likely reflecting DAG‐induced opening of sensitized channels. Interestingly, sensitization of TRPC3 channels by cis‐OptoDArG at levels below threshold for channel opening was not readily reversed by blue light illumination. This is in striking contrast to the observed photolipid‐mediated conductance activation process and suggests that two distinct conformational states, that is, a sensitized closed and an activated state, are induced by cis‐OptoDArG. It is tempting to speculate that the sensitizing interaction of cis‐OptoDArG with TRPC3 stabilizes its cis conformation and impedes its conversion into the trans conformation by blue light. Such a retrograde effect of the target protein on photoisomerization of the agonist needs to be tested in further studies.

Two lines of evidence support a novel concept in which DAG can occupy the L2 site within the closed TRPC3 pore domain to generate a sensitized state of the channel: i) MD simulations using the currently available TRPC3 structure in its closed conformation (Fan *et al*, 2018) revealed efficient DAG binding to L2, and ii) lipid‐clamp electrophysiology revealed that OptoDArG at levels below the threshold for channel activation prepares closed channels to open significantly faster during subsequent “above‐threshold” DAG photocycling. It remains to be clarified if full activation of TRPC3 involves additional DAG‐induced conformational changes within the L2 domains of the tetramer. We suggest that sub‐stoichiometric, partial DAG lipidation of the closed tetramer facilitates the channel opening by further occupancy of vacant L2 coordination sites. This hypothesis is consistent with the observed double to triple occupancy of closed tetramers by SAG in the MD simulations. Nonetheless, to this end, we cannot exclude that full activation of the channel involves additional interactions of DAG outside of L2.

As the kinetics of the sensitization step cannot be determined from these experiments, we set out to further test the role of L2 by determining the threshold level required for sensitization of WT and mutant channels.

### Mutation of L2 (G652A) reduces the threshold for sensitization of TRPC3 by cis OptoDArG

**Figure 5:**
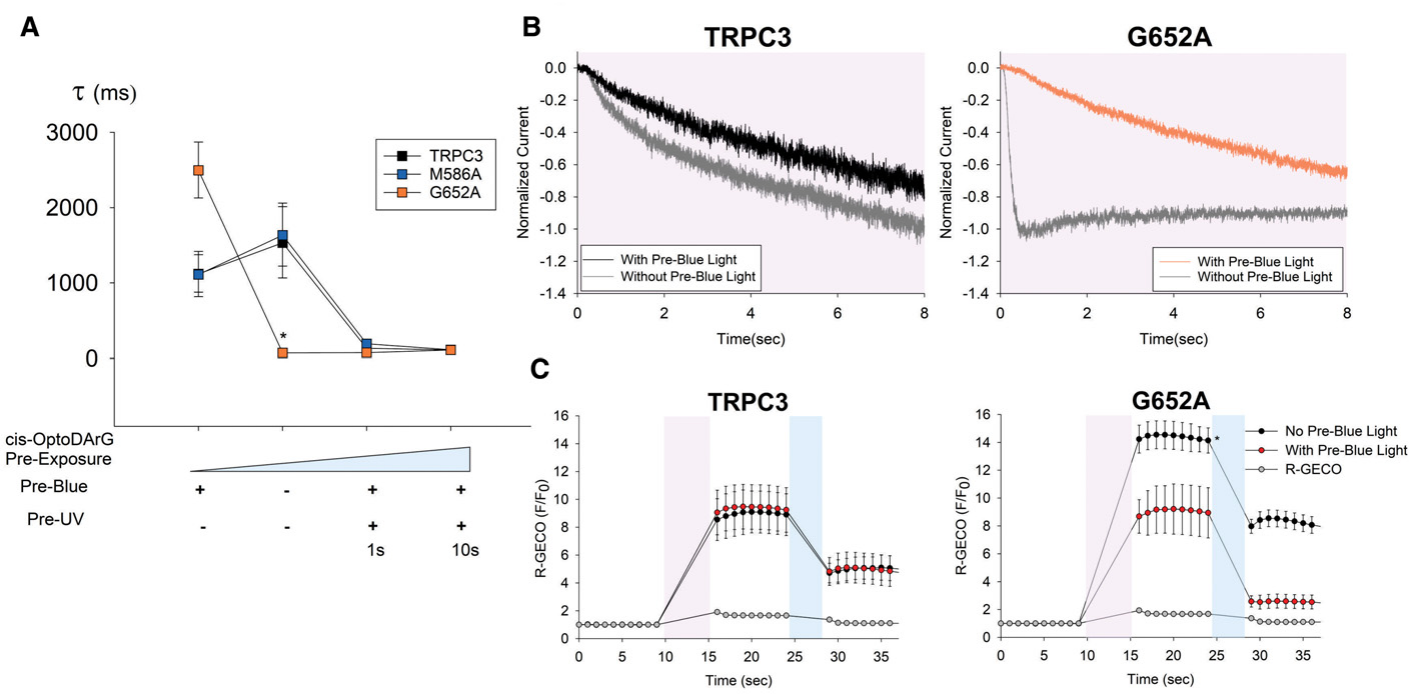
A mutation of L2 (G652A) enables sensitization of TRPC3 at low levels of cis OptoDArG Plot of the time constants of current activation (Tau, τ) for TRPC3‐WT, M586A and G652A channels at different experimental settings and levels of pre‐exposure to cis‐OptoDArG. Number of biological repetitions ≥ 6. Data are mean ± SEM; two‐tailed *t*‐test or Mann–Whitney test were applied; *P < 0.05.Representative traces showing the normalized inward current activation of TRPC3‐WT and G652A induced by OptoDArG (20 μM) in the absence and presence of Pre‐Blue illumination.Time courses of Ca^2+^ ‐sensitive R‐GECO fluorescence during OptoDArG (20 μM) photoactivation (100% UV intensity; 365 nm for 5 s (violet), blue light: 430 nm for 3 s (blue)) in the absence and presence of Pre‐Blue light for overexpressed TRPC3‐WT and G652A in HEK293 cells. Number of biological repetitions ≥ 6. Data are mean ± SEM; two‐tailed *t*‐test or Mann–Whitney test were applied; *P < 0.05.

To delineate the required threshold level of OptoDArG for TRPC3 sensitization, we plotted the time constants for channel activation (Tau “ON”), as it represents a reliable indicator of sensitization for conditions of different levels of DAG pre‐exposure (Fig 5A). After a 20‐s illumination by blue light and no optical intervention (+ Pre‐Blue), the bath solution lacked significant levels of cis activating OptoDArG. Experiments, in which channels were activated without a preceding blue light illumination (− Pre‐Blue), were considered to expose the channels to a significant but low level of cis OptoDArG. Pre‐exposure of channels to the lipid activator was further enhanced in experiments introducing a short and moderate UV pre‐illumination (Pre‐UV, 1 s, 30%), and a maximum pre‐exposure to the activating DAG was assumed for a preceding, fully activating pulse (Pre‐UV, 10 s, 100%). As evident from Fig 5A, G652A mutant channels were significantly sensitized already when omitting the blue light pre‐illumination (− Pre‐Blue), while TRPC3 WT as well as the M586A mutant required a short pre‐illumination with UV light (1 s; 30%). These experiments demonstrate that the L2 mutation G652A significantly shifts the threshold and therefore the concentration dependence of TRPC3 channels for DAG‐mediated sensitization. Promotion of TRPC3 sensitization by the L2 mutation, as illustrated at the membrane current level in Fig 5B, was corroborated at the level of Ca^2+^ entry and of cytoplasmic Ca^2+^ levels as shown in Fig 5C.

**Figure EV3:**
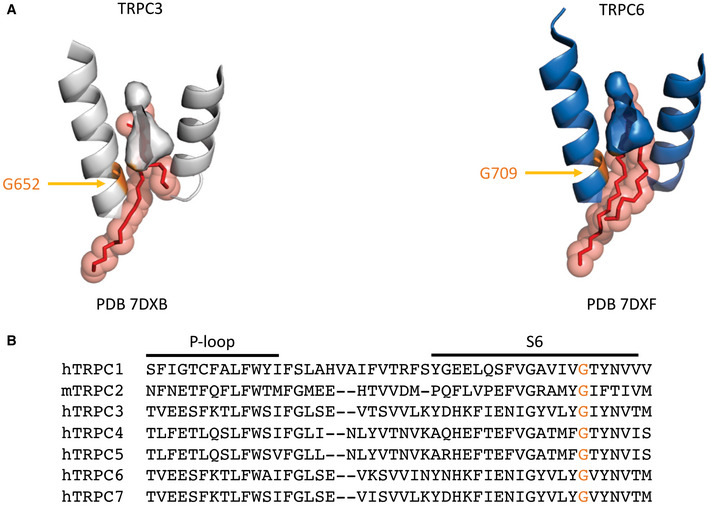
Highly conserved DAG accommodation site in TRPC channels L2 binding site structure of TRPC3 (hTRPC3 PDB ID: 7DXB; Guo *et al*, 2022) and hTRPC6 (PDB ID: 7DXF; Guo *et al*, 2022). G652 in TRPC3 and homologous G709 in TRPC6 highlighted orange.Gly residue involved in DAG accommodation in L2 site in TRPC3 aligned with other TRPCs and highlighted orange. Sequence alignment of TRPCs according to Fan *et al* (2018).

In summary, our results demonstrate the ability of DAG to interact with L2 of TRPC3 in its closed conformation and to generate a sensitized closed state that enables fast and efficient channel opening during subsequent activating stimuli. The presence of a prominent DAG interaction within L2 in closed TRPC channels is consistent with a recently reported structure of TRPC3 (Guo *et al*, 2022) and the related TRPC5 channel, which was resolved in a closed state harboring DAG within L2 (Song *et al*, 2021; Storch *et al*, 2021). Our current data support the concept of a lipid coordination site, which is conserved within the pore domain of TRPC channels (L2; Fig EV3A and B) and is pivotal for DAG‐mediated control over channel functions. Moreover, our Ca^2+^ imaging experiments suggest DAG‐induced TRPC3 sensitization as a relevant mechanism to enable high efficiency of coupling between TRPC3 Ca^2+^ entry and certain Ca^2+^ sensors by generation of distinct spatiotemporal Ca^2+^ signal patterns.

## Materials and Methods

### System preparation

The cryo‐EM structure of TRPC3 (PDB: 6cud (Fan *et al*, 2018)) was obtained from the Protein Data Bank. Missing residues and atoms were modeled using Modeller 9.24 (Webb & Sali, 2016). The resulting TRPC3 structure was truncated with PDB‐tools to the transmembrane domain (residue 322 to residue 699, isoform 3 numbering) and uploaded to the Position of Proteins in Membranes (PPM) server (Lomize *et al*, 2012; Rodrigues *et al*, 2018) in order to determine its position in the membrane. The mutant G652A channel was generated by using the Pymol Mutagenesis Wizard (The PyMOL Molecular Graphics System, Version 1.2r3pre, Schrödinger, LLC.). The martinize.py script (version 2.4; http://cgmartini.nl/images/tools/martinize/martinize‐2.4/martinize.py) was used to convert TRPC3 to a coarse‐grained representation and insane.py (http://www.cgmartini.nl/images/tools/insane/insane.py) was used to embed it in a lipid bilayer according to the concentrations found in Appendix Table S2. These concentrations were selected according to previously published literature (Hedger *et al*, 2019). The system was solvated, including 10% anti‐freeze water particles, and NaCl was added to a total concentration of 150 mM. The systems have size of ca. 24 × 24 × 12 nm and contain approximately 1300 lipids.

### Simulation parameters

Simulations were run using GROMACS version 2020.1 with the Martini 2.2 forcefield for proteins and Martini 2.0 forcefield for lipids (Marrink *et al*, 2004, 2007; Van Der Spoel *et al*, 2005; de Jong *et al*, 2013; Abraham *et al*, 2015; Wassenaar *et al*, 2015). The simulations were conducted at a temperature of 310 K, maintained using the velocity rescaling thermostat, and a pressure of 1 bar, maintained using the Parrinello‐Rahman barostat. The Reaction‐Field algorithm was used for electrostatic interactions with a cut‐off of 1.1 nm, and a single cutoff of 1.2 nm was used for Van der Waals interactions. A time step of 20 fs was used, and coordinates were saved every 5000 steps. Neighbor searching was performed every 20 steps.

The 20 µs production runs were performed in the presence of position restraints on the backbone atoms of the protein, with a force constant of 10 kJ mol^−1^ nm^−2^. For the 2% DAG, after 20 µs, the position restraints were removed and the simulation was continued for a further 20 µs, with the secondary and tertiary structures of the protein constrained by the ElNeDyn elastic network (Periole *et al*, 2009).

### Analysis

Analysis was carried out using GROMACS version 2020.1 analysis tools, the MDAnalysis package and VMD (version 1.9.3) analysis scripts (Humphrey *et al*, 1996; Michaud‐Agrawal *et al*, 2011; Gowers *et al*, 2016). In more detail, VMD analysis scripts written in‐house were used to calculate the time‐resolved number of lipid molecules within 0.6 nm of TRPC3. The density maps were calculated using the VMD volmap plugin, with a resolution of 0.1 nm. For the breakdown of lipid contacts at the L1 and L2 binding sites, “gmx select” was used to identify the CG beads within 0.6 nm of the L1 and L2 amino acids. Graphs were plotted using Matplotlib and images were produced using VMD.

### Cells, DNA constructs, and reagents

HEK293—Human Embryonic Kidney Cells (CLS 300192), hTRPC3 (Q13507‐3)‐peYFP‐C1, hTRPC3‐(C350A)‐peYFP‐C1, hTRPC3‐(I394F)‐peYFP‐C1, hTRPC3‐(N560S)‐peYFP‐C1, hTRPC3‐(M586A)‐peYFP‐C1, hTRPC3‐(Y648F)‐peYFP‐C1, hTRPC3‐(G652A)‐peYFP‐C1, CMV‐R‐GECO1.2 (#45494) and mTagRFP‐Membrane‐1 (Addgene #57992) were obtained from Addgene, and OptoDArG was synthesized at Bio‐Techne (Bristol, GB).

### Cell culture and transfection

Human embryonic kidney 293 (HEK293) cells were cultured in Dulbecco’s modified Eagle medium (DMEM, D6429, Invitrogen,) with 10% supplement of fetal bovine serum (FBS), streptomycin (100 μg/ml), penicillin (100 U/ml), l‐glutamine (2 mM/l) and HEPES (10 mM/l) at constant 37°C temperature and 5% CO_2_ level. Cells were authenticated by STR and regular tests were performed to confirm lack of contamination with mycoplasms. For transfection, the media was aspirated from the cell culture flask. HEK293 cells were rinsed with PBS. Cells were incubated with accutase (250–500 μl) for 5 min at 37°C. Detached cell suspension was mixed with fresh DMEM in two times the volume of accutase. 1 × 10^5^ cells suspension was centrifuged at 300 *g* for 2 min. Supernatant was discarded, and the cell pellet was suspended in serum‐free medium (60 μl). Cells were transiently transfected with 1 μg plasmid DNA using PolyJet (SignaGen Laboratories) according to the manufacturer’s protocol. Cells were seeded on 6 × 6 mm glass coverslips, and the medium was changed after 6‐h incubation.

### Electrophysiology

Transfected HEK293 cells were seeded on glass coverslips the day before the experiments. After 24 h, coverslips were mounted in a perfusion chamber on an inverted microscope (Zeiss Axiovert 200 M, Germany) with 40×/0.75 objective. CoolLED Pw‐300Ultra was used as an excitation source. Transfected cells were detected by illumination at 490 nm wavelength. Patch‐clamp recordings were performed in whole‐cell configuration using an Axopatch 200B amplifier (Molecular Devices) connected with a Digidata‐1550B Digitizer (Axon Instruments). Signals were low‐pass filtered at 2 kHz and digitized with 8 kHz. The application of linear voltage‐ramp protocols ranging from −130 to +80 mV (holding potential 0 mV) was controlled by Clampex 10.7 (Axon Instruments) software. Current densities at −90 and +70 mV were plotted against time and normalized by capacitance. For the pharmacological measurements, cells were perfused with 200 µM carbachol in ECS at room temperature. For photopharmacological measurements, coverslips were transferred in a perfusion chamber filled with 20 µM OptoDArG, then cells were repetitively illuminated with UV (365 nm) and blue (430 nm) light. The voltage‐clamp extracellular solutions (ECS) contained (in mM): 140 NaCl, 10 HEPES, 10 Glucose, 2 MgCl_2_, 2 CaCl_2_, pH adjusted to 7.4 with NaOH. Pipette solution (ICS) contained (in mM): 150 cesium methanesulfonate, 20 CsCl, 15 HEPES, 5 MgCl_2_, 3 EGTA, titrated to pH 7.3 with CsOH. Thin‐wall capillary pipettes made by borosilicate glass with filament (Harvard Apparatus) were pulled to a resistance of 3–4 MΩ.

### Ca^2+^ imaging

For Ca^2+^ imaging, HEK293 cells were co‐transfected with YFP‐TRPC3 and mCherry‐R‐GECO constructs and seeded on coverslips the day prior to the experiments. For detection of Ca^2+^ changes in cytoplasm during the experiments, cells were illuminated with 570 nm. Cells during the experiment were kept in ECS containing (mM): 140 NaCl, 10 HEPES, 10 glucose, 2 CaCl_2_, and 2 MgCl_2_ at pH 7.4 (adjusted with NaOH). Cells which were seeded on the cover slip were transferred to a bath with 20 μM OptoDArG in ECS on an inverted microscope with 40 × 1.3 N.A. oil‐immersion objective (Olympus IX71) at room temperature. Photoisomerization of the OptoDArG was triggered by exposure to 5 s illumination periods at 365 nm (UV light) or 3 s periods at 430 nm (Blue light). In order to measure [Ca^2+^]_i_ with R‐GECO, cells were illuminated every second at 577 nm. F/F_0_ is calculated by normalization of the cell fluorescence to background fluorescence which is drawn in an area without any cells.

### Statistics

Data analysis and graphical display were performed using Clampfit 11 (Axon Instruments) and SigmaPlot 14.0 (Systat Software Inc.). Data are presented as mean values ± SEM. For normal distributed values (confirmed by Shapiro–Wilk test), Student’s two‐sample *t*‐test or paired *t*‐test were used to analyze the statistical significance. Equality of variances was tested by Levene´s test and ANOVA was performed when appropriate. For non‐normally distributed values, Mann–Whitney rank sum test was applied. In general, differences were considered significant at *P* < 0.05.

### Curve fitting

The fitting of the model function to measured data was implemented in Python V3.9.1 (van Rossum & Drake, 2009) utilizing the scientific computing packages NumPy V1.19.5 (Harris *et al*, 2020) and SciPy V1.6.0 (Virtanen *et al*, 2020). A curve fitting method based on a non‐linear least squares problem was applied to optimize the set of model parameters, such that the corresponding model function fits the measured data with and time points for. To determine the optimal set of parameters, the following least squares problem was solved with respect to the parameter set and with component wise lower and upper bounds on the parameters, denoted by and respectively (Appendix Table S3). Since the parameters are bounded, a suitable method for solving the least squares problem is needed. In addition, the choice of the loss function influences the robustness of the corresponding least squares problem. For our application, the loss function, which is a smooth approximation of the standard loss function, in combination with a Trust Region Reflective algorithm, see (Moré, 1978; Branch *et al*, 1999), showed the most robust behavior.

## Author contributions

**Hazel Erkan‐Candag:** Data curation; Formal analysis; Investigation; Writing—original draft. **Amy Clarke:** Data curation; Formal analysis; Investigation; Writing—original draft. **Oleksandra Tiapko:** Data curation; Formal analysis; Investigation; Methodology; Writing—review and editing. **Mathias AF Gsell:** Formal analysis; Methodology; Writing—review and editing. **Thomas Stockner:** Conceptualization; Supervision; Funding acquisition; Methodology; Writing—review and editing. **Klaus Groschner:** Conceptualization; Supervision; Funding acquisition; Writing—original draft; Project administration; Writing—review and editing.

## Disclosure and competing interests statement

The authors declare that they have no conflict of interest.

## Supporting information



Appendix

Expanded View Figures PDF

## Data Availability

This study includes no data deposited in external repositories. Additional data related to this paper may be requested from the corresponding authors.
