# Supplementary material for: Diacylglycerols interact with the L2 lipidation site in TRPC3 to induce a sensitized channel state
Source: EMBO Rep. 2022 May 23;23(7):e54276. doi: 10.15252/embr.202154276 (PMC9253791; doi:10.15252/embr.202154276)
Supplement: Supplementary file 1 — Appendix [file EMBR-23-e54276-s001.pdf]

Diacylglycerols interact with the L2 lipidation site in TRPC3 to induce a sensitized channel state

Hazel Erkan-Candag [1,#], Amy Clarke [2,#], Oleksandra Tiapko [1], Mathias A.F. Gsell [1], Thomas Stockner [2,*] and Klaus Groschner [1,]**

[1] Gottfried-Schatz-Research-Center – Biophysics, Medical University of Graz, Graz, Austria
[2] Institute of Pharmacology, Medical University of Vienna, Vienna, Austria

*Corresponding author. Tel: +43 1 40160 31215; E-mail: thomas.stockner@meduniwien.ac.at;

**Corresponding author. Tel.: +43 316 385 71500; E-mail: klaus.groschner@medunigraz.at

# Authors contributed equally

Appendix Table of Contents

Appendix Figures:

Appendix Figure S1. Fluorescence images of WT and mutant TRPC3 membrane targeting……………………………………………………………………………………2

Appendix Table:

Appendix Table S1. Speed of current activation and power with repetitive photostimulation...…………………………………………………………………………4

Appendix Table S2. Membrane systems used in this paper……………………………...…5

Appendix Table S3. Lower and upper bounds of the parameter set for curve fitting..……6

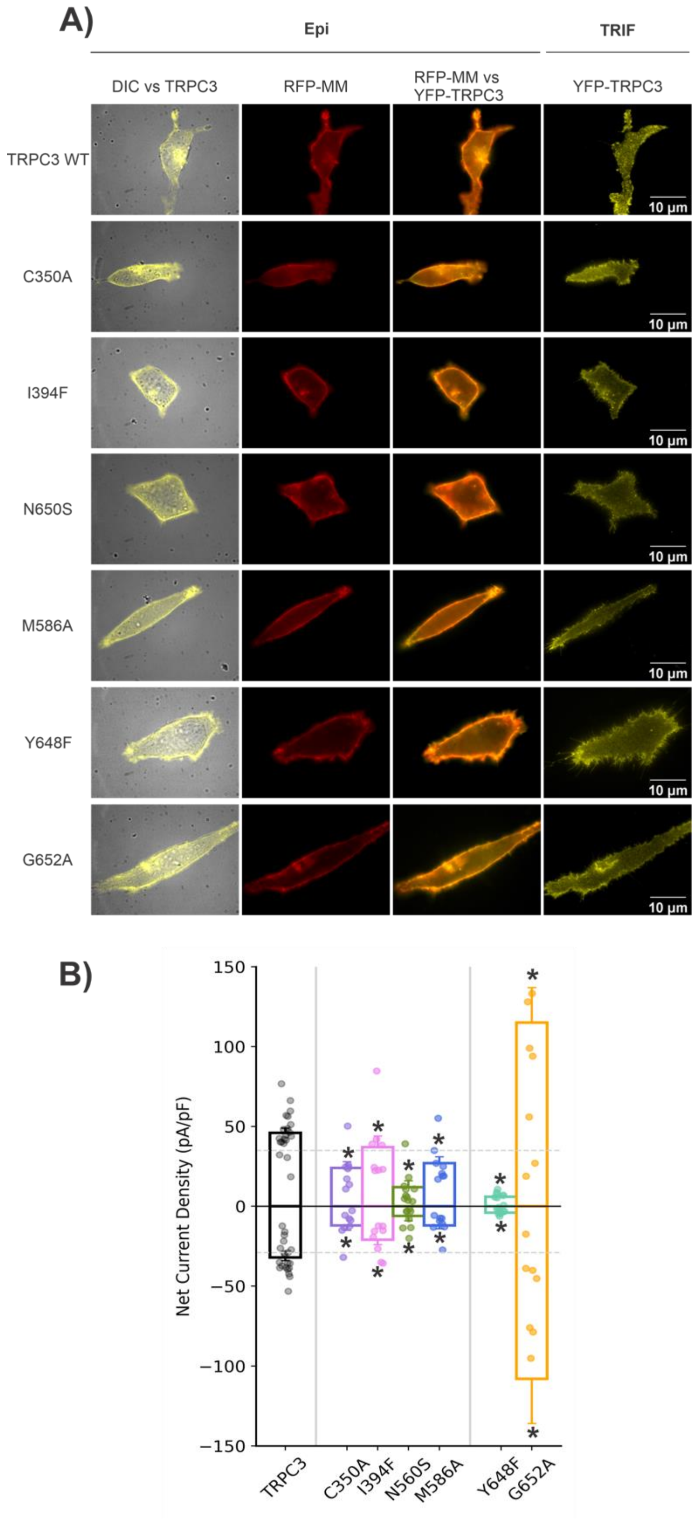

Appendix Figure S1 - Fluorescence images of WT and mutant TRPC3 membrane targeting.

A. representative epifluorescence (EF) and TIRF images of single cells expressing TRPC3 WT and TRPC3 mutants (L1 Mutants: C350A; I394F; N560S; M586A; L2 Mutants: Y648F; G652A). N = number of cells measured > 6 for each of the construct; scale bar = 10 µm.

B. Mean net current densities of GSK (10 μM)-stimulated inward and outward currents through TRPC3-WT (black) and mutant channels (color-coded as indicated) expressed in HEK293 cells. Values from individual experiments are shown for each of the columns (circles). Number of biological repetitions for each condition ≥ 6. Data are mean ± S.E.M; two-tailed t-test or Mann-Whitney test were applied between TRPC3-WT and mutants; *$P < 0.05$.

Constructs	1st UV ON τ ± SEM	2nd UV ON τ ± SEM	1st UV ON N (Power)	2nd UV ON N (Power)
TRPC3	1127 ± 250	128 ± 7	3.6 ± 0,7	2.0 ± 0,2
C350A	1130 ± 328	96 ± 20	3.6 ± 1,4	3.3 ± 0,5
I394F	1106 ± 185	113 ± 13	3.6 ± 1,6	2.1 ± 0,3
N650S	897 ± 153	106 ± 16	3.5 ± 0,5	3.3 ± 0,9
M586A	1029 ± 422	121 ± 8	3.2 ± 0,7	2.8* ± 0,3
Y648F	990 ± 292	44* ± 8	2.7 ± 0,9	9.9* ± 1,2
G652A	2918* ± 647	118 ± 3	3.6 ± 1	2.5 ± 0,5

Appendix Table S1 - Speed of current activation and power with repetitive photostimulation.

Number of biological repetitions ≥ 6. Data are mean ± S.E.M.; two-tailed t-test or Mann-Whitney test were applied between TRPC3-WT and mutants; *P < 0.05.

System	Lipid ratios, inner leaflet (%)	Lipid ratios, outer leaflet (%)	Length (μs)
Control	Chol (30), PC (20), PE (35), PS (15)	Chol (30), PC (60), PE (10)	20
2% DAG (SAG)	DAG (2), Chol (28), PC (20), PE (35), PS (15)	Chol (30), PC (60), PE (10)	40
10% DAG (SAG)	DAG (10), Chol (20), PC (20), PE (35), PS (15)	Chol (30), PC (60), PE (10)	20

Appendix Table S2 - Membrane systems used in this paper.

The abbreviations used in this table are: PC (phosphatidylcholine, POPC 1-palmitoyl-2-oleoyl-sn-glycero-3-phosphatidylcholine), Chol (cholesterol, CHOL), PE (phosphatidylethanolamine, POPE 1-palmitoyl-2-oleoyl-sn-glycero-3-phosphatidylethanolamine), PS (phosphatidylserine, POPS 1-palmitoyl-2-oleoyl-sn-glycero-3-phosphatidylserine), and DAG (diacylglycerol, specifically stearoyl-arachidonoyl glycerol, PADG stearoyl-arachidonoyl-diacylglycerol).

lb				*ub*
$-\infty$	$<$	A	$\leq$	0
0	$\leq$	S	$<$	$+\infty$
10	$\leq$	T	$<$	$+\infty$
1	$\leq$	P	$<$	$+\infty$
$-\infty$	$<$	K	$\leq$	0
$-\infty$	$<$	C	$<$	$+\infty$

Appendix Table S3 - Lower and upper bounds of the parameter set for curve fitting.
