# Supplementary material for: Diacylglycerols interact with the L2 lipidation site in TRPC3 to induce a sensitized channel state
Source: EMBO Rep. 2022 May 23;23(7):e54276. doi: 10.15252/embr.202154276 (PMC9253791; doi:10.15252/embr.202154276)
Supplement: Supplementary file 2 — Expanded View Figures PDF [file EMBR-23-e54276-s003.pdf]

Expanded View Figures

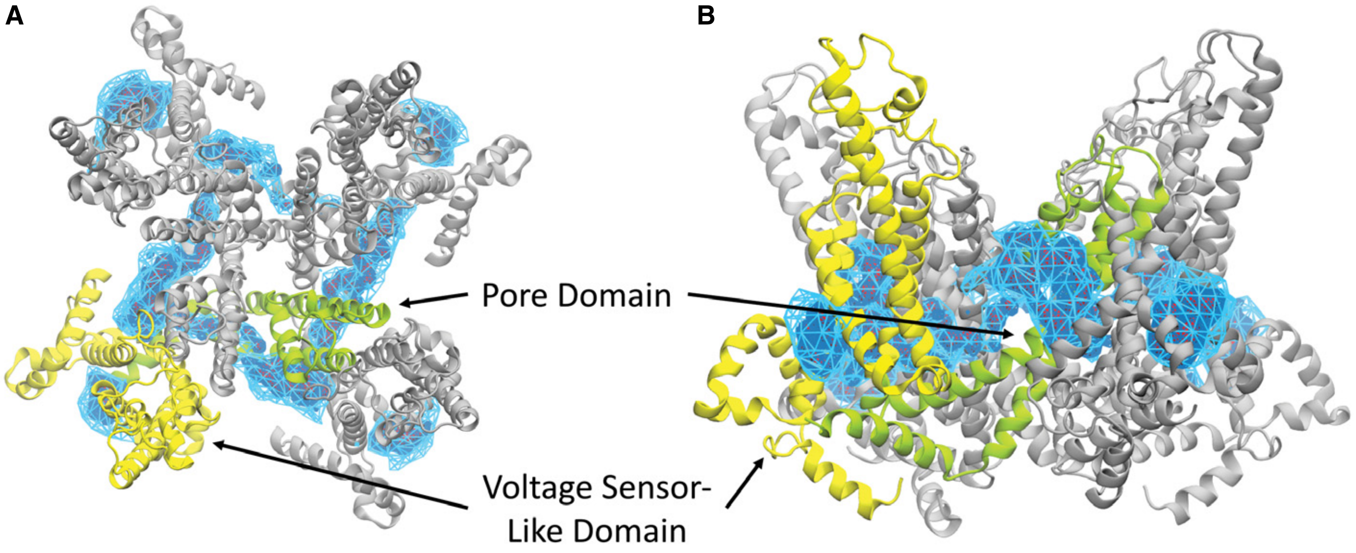

Figure EV1. Three-dimensional density maps showing DAG interaction with TRPC3.

A, B TRPC3 is shown in gray, with one subunit highlighted in yellow (voltage sensor-like domain) and green (pore domain). Blue represents DAG density. (A) Top view (B) Side view.

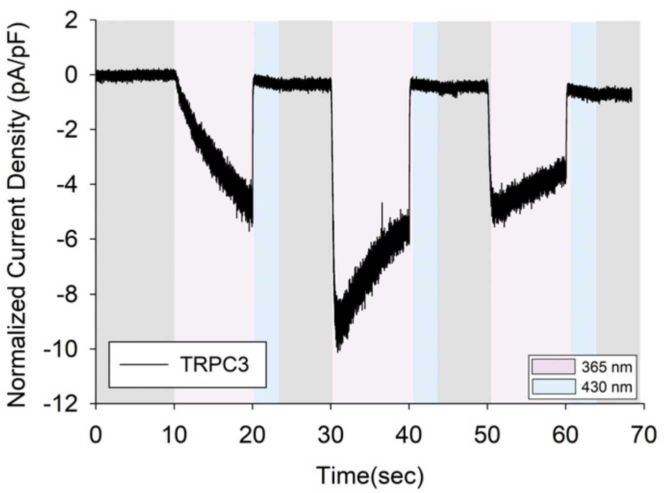

Figure EV2. Time course of WT TRPC3 during 3 repetitive activating photocycles.

Representative recordings showing the inward currents induced by 3 repetitive photoactivation of OptoDArG (20 μM) in a whole-cell, gap-free recording (holding potential: − 40 mV, normalized by capacitance) in TRPC3-WT-expressing HEK293 cells. 100% intensity of UV: 365 nm for 10 s (violet), blue light: 430 nm for 10 s (blue).

 © 2022 The Authors

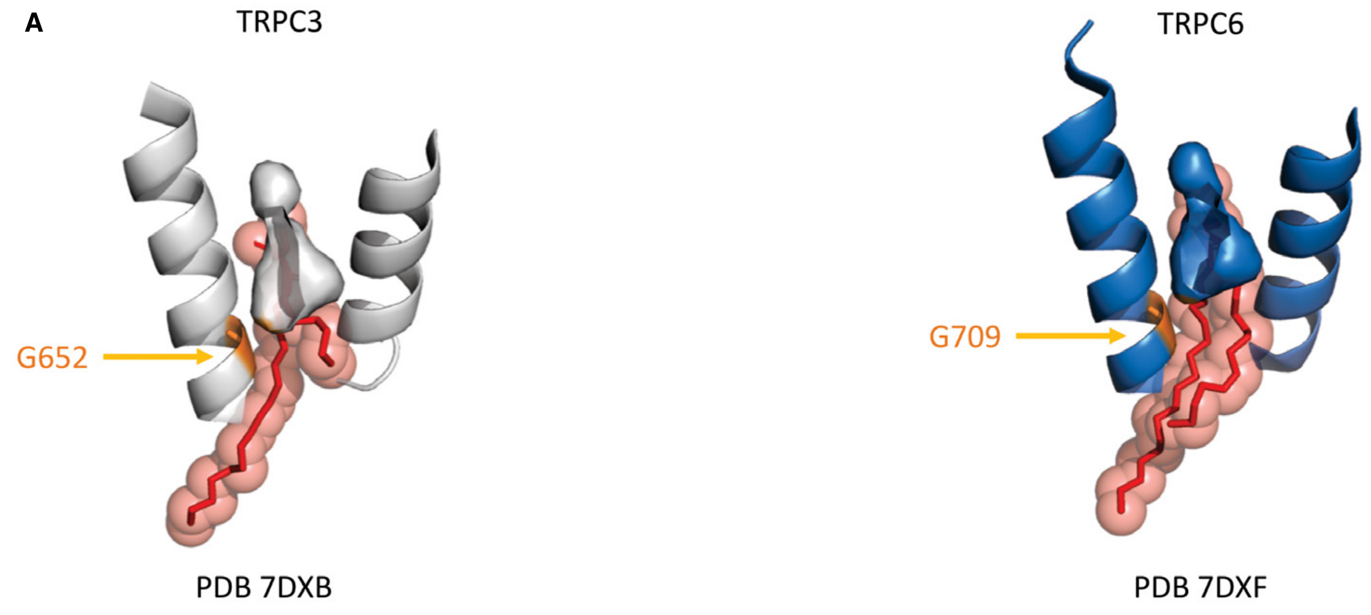

B

```
               P-loop                          S6
hTRPC1  SFIGTCFALFWYIFSLAHVAIFVTRFSYGEELQSFVGAVIVGTYNVVV
mTRPC2  NFNETFQFLFWTMFGMEE--HTVVDM-PQFLVPEFVGRAMYGIFTIVM
hTRPC3  TVEESFKTLFWSIFGLSE--VTSVVLKYDHKFIENIGYVLYGIYNVTM
hTRPC4  TLFETLQSLFWSIFGLI--NLYVTNVKAQHEFTEFVGATMFGTYNVIS
hTRPC5  TLFETLQSLFWSVFGLL--NLYVTNVKARHEFTEFVGATMFGTYNVIS
hTRPC6  TVEESFKTLFWAIFGLSE--VKSVVINYNHKFIENIGYVLYGVYNVTM
hTRPC7  TVEESFKTLFWSIFGLSE--VISVVLKYDHKFIENIGYVLYGVYNVTM
```

Figure EV3. Highly conserved DAG accommodation site in TRPC channels.

A L2 binding site structure of TRPC3 (hTRPC3 PDB ID: 7DXB; Guo *et al*, 2022) and hTRPC6 (PDB ID: 7DXF; Guo *et al*, 2022). G652 in TRPC3 and homologous G709 in TRPC6 highlighted orange.

B Gly residue involved in DAG accommodation in L2 site in TRPC3 aligned with other TRPCs and highlighted orange. Sequence alignment of TRPCs according to Fan *et al* (2018).
