# Supplementary material for: Diacylglycerols interact with the L2 lipidation site in TRPC3 to induce a sensitized channel state
Source: EMBO Rep. 2022 May 23;23(7):e54276. doi: 10.15252/embr.202154276 (PMC9253791; doi:10.15252/embr.202154276)
Supplement: Supplementary file 3 [file EMBR-23-e54276-s002.pdf]

EMBO reports

Diacylglycerols interact with the L2 lipidation site in TRPC3 to induce a sensitized channel state

Hazel Erkan-Candag, Amy Clarke, Oleksandra Tiapko, Mathias Gsell, Thomas Stockner, and Klaus Groschner
DOI: 10.15252/embr.202154276

Corresponding author(s): Klaus Groschner (klaus.groschner@medunigraz.at)

Review Timeline:

Submission Date:	4th Nov 21
Editorial Decision:	1st Dec 21
Revision Received:	1st Mar 22
Editorial Decision:	5th Apr 22
Revision Received:	11th Apr 22
Accepted:	25th Apr 22

Editor: Martina Rembold

Transaction Report:

(Note: With the exception of the correction of typographical or spelling errors that could be a source of ambiguity, letters and reports are not edited. Depending on transfer agreements, referee reports obtained elsewhere may or may not be included in this compilation. Referee reports are anonymous unless the Referee chooses to sign their reports.)

1st Editorial Decision 1st Dec 2021

Dear Prof. Groschner

Thank you for the submission of your research manuscript to our journal. We have now received the full set of referee reports that is copied below.

As you will see, the referees acknowledge that the findings are potentially interesting, but they also raise a number of important criticisms that need to be addressed. The fact that the channel mutations have different effects on currents induced by carbachol or by OptoDArG was noted by referee 2 and 3 and this discrepancy needs to be clarified. In general, the different L1 and L2 mutants need to be better characterized in terms of channel functionality and DAG binding and their proposed effects on gating be substantiated and clarified.

I realize that addressing all these concerns will result in a major revision with an uncertain outcome. But given the potential interest of your findings and the constructive comments, I would like to give you the chance to revise your manuscript with the understanding that the referee concerns (as detailed above and in their reports) must be fully addressed and their suggestions taken on board. Please address all referee concerns in a complete point-by-point response. Acceptance of the manuscript will depend on a positive outcome of a second round of review. It is EMBO reports policy to allow a single round of revision only and acceptance or rejection of the manuscript will therefore depend on the completeness of your responses included in the next, final version of the manuscript.

Revised manuscripts should be submitted within three months of a request for revision; they will otherwise be treated as new submissions. Please contact us if a 3-months time frame is not sufficient for the revisions so that we can discuss the revisions further.

***IMPORTANT NOTE: we perform an initial quality control of all revised manuscripts before re-review. Your manuscript will FAIL this control and the handling will be DELAYED if the following APPLIES:

1) A data availability section is missing.
2) Your manuscript contains error bars based on n=2. Please use scatter blots showing the individual datapoints in these cases. The use of statistical tests needs to be justified.

When submitting your revised manuscript, please carefully review the instructions that follow below. Failure to include requested items will delay the evaluation of your revision.***

When submitting your revised manuscript, we will require:

1) a .docx formatted version of the manuscript text (including legends for main figures, EV figures and tables). Please make sure that the changes are highlighted to be clearly visible.

2) individual production quality figure files as .eps, .tif, .jpg (one file per figure).
Please download our Figure Preparation Guidelines (figure preparation pdf) from our Author Guidelines pages https://www.embopress.org/page/journal/14693178/authorguide for more info on how to prepare your figures.

3) a .docx formatted letter INCLUDING the reviewers' reports and your detailed point-by-point responses to their comments. As part of the EMBO Press transparent editorial process, the point-by-point response is part of the Review Process File (RPF), which will be published alongside your paper.

4) a complete author checklist, which you can download from our author guidelines (). Please insert information in the checklist that is also reflected in the manuscript. The completed author checklist will also be part of the RPF.

5) Please note that all corresponding authors are required to supply an ORCID ID for their name upon submission of a revised manuscript (). Please find instructions on how to link your ORCID ID to your account in our manuscript tracking system in our Author guidelines
()

6) We replaced Supplementary Information with Expanded View (EV) Figures and Tables that are collapsible/expandable online. A maximum of 5 EV Figures can be typeset. EV Figures should be cited as 'Figure EV1, Figure EV2" etc... in the text and their respective legends should be included in the main text after the legends of regular figures.

- For the figures that you do NOT wish to display as Expanded View figures, they should be bundled together with their legends in a single PDF file called *Appendix*, which should start with a short Table of Content. Appendix figures should be referred to in

the main text as: "Appendix Figure S1, Appendix Figure S2" etc. See detailed instructions regarding expanded view here:

- Additional Tables/Datasets should be labeled and referred to as Table EV1, Dataset EV1, etc. Legends have to be provided in a separate tab in case of .xls files. Alternatively, the legend can be supplied as a separate text file (README) and zipped together with the Table/Dataset file.

7) Please note that a Data Availability section at the end of Materials and Methods is now mandatory. In case you have no data that requires deposition in a public database, please state so instead of refereeing to the database.
See also < https://www.embopress.org/page/journal/14693178/authorguide#dataavailability>). Please note that the Data Availability Section is restricted to new primary data that are part of this study.

8) Figure legends and data quantification:
The following points must be specified in each figure legend:
- the name of the statistical test used to generate error bars and P values,
- the number (n) of independent experiments (please specify technical or biological replicates) underlying each data point,
- the nature of the bars and error bars (s.d., s.e.m.)
- If the data are obtained from n {less than or equal to} 2, use scatter blots showing the individual data points.
Discussion of statistical methodology can be reported in the materials and methods section, but figure legends should contain a basic description of n, P and the test applied.
See also the guidelines for figure legend preparation:
https://www.embopress.org/page/journal/14693178/authorguide#figureformat
- Please also include scale bars in all microscopy images.

9) We would also encourage you to include the source data for figure panels that show essential data. Numerical data should be provided as individual .xls or .csv files (including a tab describing the data). For blots or microscopy, uncropped images should be submitted (using a zip archive if multiple images need to be supplied for one panel). Additional information on source data and instruction on how to label the files are available .

10) Our journal encourages inclusion of *data citations in the reference list* to directly cite datasets that were re-used and obtained from public databases. Data citations in the article text are distinct from normal bibliographical citations and should directly link to the database records from which the data can be accessed. In the main text, data citations are formatted as follows: "Data ref: Smith et al, 2001" or "Data ref: NCBI Sequence Read Archive PRJNA342805, 2017". In the Reference list, data citations must be labeled with "[DATASET]". A data reference must provide the database name, accession number/identifiers and a resolvable link to the landing page from which the data can be accessed at the end of the reference. Further instructions are available at .

11) As part of the EMBO publication's Transparent Editorial Process, EMBO reports publishes online a Review Process File to accompany accepted manuscripts. This File will be published in conjunction with your paper and will include the referee reports, your point-by-point response and all pertinent correspondence relating to the manuscript.

You are able to opt out of this by letting the editorial office know (emboreports@embo.org). If you do opt out, the Review Process File link will point to the following statement: "No Review Process File is available with this article, as the authors have chosen not to make the review process public in this case."

We would also welcome the submission of cover suggestions, or motifs to be used by our Graphics Illustrator in designing a cover.

I look forward to seeing a revised version of your manuscript when it is ready. Please let me know if you have questions or comments regarding the revision.

Yours sincerely,

Martina Rembold, PhD
Senior Editor
EMBO reports

****

Referee #1:

Erkan-Candag et al. analyzed lipid binding to the L1 and L2 domains of TRPC3 channels by structure-guided mutagenesis, electrophysiological recordings and molecular dynamics simulations. Diacylglycerol a known activator of TRPC3 rapidly

interacted and sensitized the channel. By usage of a photoswitchable DAG-probe, authors were able to show potentiation of TRPC3 currents during repetitive activation. Mutations in L2 had a higher impact on DAG interaction with the G625A mutation shifting sensitization to very low levels of DAG. The authors use state-of-the-art technologies to provide strong evidence for a novel concept of DAG binding. I have only four minor remarks.

(1) Supplementary Figure 2: Please provide overlays of fluorescent and differential interference contrast (DIC) images for the epifluorescence pictures.
(2) Please discuss the relevance of your conclusions for other primarily DAG-sensitive members of the TRPC family. Are mutated residues identical and in a similar location in TRPC2, TRPC6 and TRPC7 channels.
(3) Figure 2 c, d: The high number of contacts of other lipids to residues in the L1 region (e.g. cholesterol) and in the L2 region (e.g. PC) are somewhat surprising. Is there any physiological relevance of these lipids in activation of TRPC3 channels?
(4) Introduction last sentence on page 1: "Interestingly, DAG-induced activation has also been observed for TRPC4 and TRPC5, but only ...". Please add the correct reference here.

Referee #2:

Members of the TRPC family of ion channels have been proposed to be directly activated by lipids including diacylglycerol (DAG), which is a lipid second messenger generated by phospholipase C activation. Recent single particle cryo-EM structures of TRPC3 revealed two lipid binding pockets, termed L1 and L2, located in distinct subregions of the transmembrane domain, however, whether and how these lipid coordination sites facilitate DAG-induced channel activation has remained elusive. In the current manuscript, Erkan-Candag et al use molecular dynamics (MD) simulations, structure-guided mutagenesis, a photo-switchable DAG analogue termed OptoDArG, and electrophysiology to probe the contribution of the L1 and L2 lipid coordination sites to DAG-mediated TRPC3 channel regulation. MD simulations with a coarse-grained TRPC3 model and varying percentages of DAG in the membrane revealed that DAG interacts with specific residues in the L1 and L2 sites. This information along with previously published data was used to guide mutagenesis of residues in the L1 and L2 sites aimed at reducing DAG binding, which were used for whole-cell patch clamp recordings. Erkan-Candag et al found that endogenously produced DAG via carbachol-indued muscarinic acetylcholine receptor activation yielded reduced activity for both L1 and L2 mutants compared to wild type (WT) with the L2 mutants being particularly striking. The authors interpreted this reduced activity as being due to reduced DAG binding at the L1 and L2 sites in these mutants. The authors then used OptoDArG, which can be activated with red light and inactivated with blue light, to have more precise, temporal control over the amount DAG available within the membrane. Patch clamping with OptoDArG showed that WT TRPC3 is sensitized after the first OptoDArG activation pulse, which was characterized by enhanced activation kinetics at the second OptoDArG activation pulse. Inconsistent with their endogenously produced DAG recordings, Erkan-Candag et al found that the L2 mutant G652A was more active than WT TRPC3 after the first pulse and more sensitized than WT TRPC3 after the second pulse, suggesting the G652A mutant is highly sensitized after exposure to DAG. This was confirmed using low intensity red light for the first pulse to activate a small fraction of OptoDArG insufficient to activate TRPC3 followed by a stronger second pulse to activate the channel. Sensitization of the G652A mutant was so strong that the authors had to pre-inactivate OptoDArG with a blue light pulse to abolish sensitization. From these collective studies with OptoDArG, the authors conclude that DAG binding to the L2 site sensitizes TRPC3 channels. Due to conflicting data with carbachol- or OptoDArG-mediated channel activity and lack of experimental data to determine how the L1 and L2 mutants affect DAG binding, the author's conclusions are not supported by their data and this manuscript is not suitable for publication at this time. I recommend reconsidering a revised manuscript after the following major points are addressed:

1. Inconsistent and contradictory results. In Figure 3, carbachol-mediated WT and mutant TRPC3 activity reveal reduced activity for L1 and L2 lipid site mutants, which was most pronounced for the L2 mutant G652A. However, Figures 4 and 5 show the G652A mutant has enhanced activity and sensitization than WT TRPC3 with the photo-switchable OptoDArG agonist. This disagreement needs to be addressed. If Figures 4 and 5 are correct, the authors have demonstrated that low levels of DAG do not sensitize WT TRPC3, and that they have built a high-affinity DAG sensor into TRPC3 with the G652A mutant. However, it appears the authors have misinterpreted this data to mean DAG sensitizes and has a high affinity for WT TRPC3 in the native L2 site, but it is difficult to evaluate their interpretations with the inconsistencies noted above.

2. Unknown effect of L1 and L2 mutants on DAG binding. The authors assume the TRPC3 L1 and L2 mutants reduce DAG binding compared to WT channels, which they infer from the reduced activity in Figure 3. Since accurate interpretation of their results critically relies on knowing how the L1 and L2 mutants, especially G562A, affect DAG binding, the authors should perform the same MD simulations using their mutant channels. A DAG binding assay would greatly strengthen their data as well.

3. Physiologically irrelevant DAG concentrations. PIP2 accounts for approximately 1% of the lipids in the plasma membrane, however, the authors used 2% and 10% DAG in their MD simulations. The authors must include an explanation for why such high concentrations of DAG were used for their MD simulations.

4. Unclear impact of L1 and L2 mutants on channel functionality. While the authors used TIRF microscopy to show L1 and L2 mutants localize to the plasma membrane, they did not confirm these mutants retain structural and functional integrity. This is

particularly important for the L2 site, which is in the pore domain that undergoes critical gating-associated conformational changes. The authors should use GSK1702934A, a selective and commercially available agonist for TRPC3 that does not interfere with DAG activation, to validate channel activity in the mutants independent of DAG.

Minor points to be addressed: The authors refer to a P-value <0.05 as a significant difference rather than statistical significance. In Figure 1b, the authors do not say what the blue and green are representing in the figure legend. In Figure 2e, I394 is mislabeled as I384. Figure 3a needs clarification for how the data in the figure was generated for the box plots. It is available in the methods, but it would be useful to readers to have that information in the figure legend.

Referee #3:

This paper attempts to address a long-standing question on how diacylglycerols gate TRPC channels. The authors used TRPC3 as an example and applied molecular dynamics, site-directed mutagenesis, electrophysiological analysis with optical activation of a photoswitchable DAG analog they have previously developed to determine the involvement of the two putative lipid binding sites on TRPC3 based on the recently described cryo-EM structures. The work was performed beautifully, and the results complement well with the available high resolution TRPC structures, which will add to our understanding of the gating mechanism of these important channels. The experimental results are interesting, but I have trouble understanding the implication of the mutations at the L2 site.
1) The effects of Y648F and G652A on currents induced by carbachol and OptoDArG are in conflict. While these mutations strongly reduced the carbachol-evoked TRPC3 current, they enhanced and accelerated the current induced by optical activation of OptoDArG. The reasons and implications of these results need to be discussed.
2) If Y648F and G652A mutations are supposed to disrupt the binding of DAG to L2, why would they facilitate channel gating by the cis OptoDArG? Is cis OptoDArG supposed to bind better to the mutant than the wild-type channel?
3) Page 4, "to affect the channel's lipidation" needs an apostrophe before "s".
4) Page 4, "This channel state persisted during further activating pulses (Suppl. Fig 2)". Is this supposed to be Suppl. Fig. 3? Also, "subsequent" may be a better word here than "further".
5) Page 5, "which displayed similarly accelerated current activation, but only minor potentiation of the maximum current amplitude". Which figure does this refer to?
6) Page 5, "At higher DAG levels, the lipid mediator presumably increases the open probability of TRPC3 by an additional DAG-induced conformational change within the channel". It is not obvious to me how this works.
7) Page 5, "presumably reflecting DAG induced opening of sensitized channels". How does this work? Does it still involve DAG binding to L2 or to a different site?
8) Page 5, "exposure is considered to lack any preexposure to cis-OptoDArG". This sentence is awkward and hard to understand.
9) Page 6, "(Fan et al.)" needs the publication year.
10) Page 6, "to be clarified if full activation of TRPC3 involves...". Which condition is considered a "full activation of TRPC3"?
11) Page 6 "striking contrast to the virtual photolipid-mediated channel activation process". What do you refer to as "the virtual photolipid-mediated channel activation process"? Is it shown in one of the figures or just an imaginary process?

Reply to Reviewers´comments:

The authors wish to thank all reviewers for the time and effort spent to help improving our manuscript.

Referee #1:

Erkan-Candag et al. analyzed lipid binding to the L1 and L2 domains of TRPC3 channels by structure-guided mutagenesis, electrophysiological recordings and molecular dynamics simulations. Diacylglycerol a known activator of TRPC3 rapidly interacted and sensitized the channel.

By usage of a photoswitchable DAG-probe, authors were able to show potentiation of TRPC3 currents during repetitive activation. Mutations in L2 had a higher impact on DAG interaction with the G625A mutation shifting sensitization to very low levels of DAG. The authors use state-of-the-art technologies to provide strong evidence for a novel concept of DAG binding. I have only four minor remarks.

The authors appreciate the positive feedback by the reviewer.

(1) Supplementary Figure 2: Please provide overlays of fluorescent and differential interference contrast (DIC) images for the epifluorescence pictures.

As suggested, we show now the overlay of DIC and epifluorescence images along with localization of a plasma membrane marker and corresponding TIRF images for the TRPC3 fusion constructs (*Appendix Figure S1*).

(2) Please discuss the relevance of your conclusions for other primarily DAG-sensitive members of the TRPC family. Are mutated residues identical and in a similar location in TRPC2, TRPC6 and TRPC7 channels.

The investigated lipid coordination sites, specifically L2, appear conserved within the primarily DAG-regulated TRPC channels. Cryo-EM studies have identified lipid densities in both L1 and L2 region of TRPC3/6 (*now shown in Figure EV3*). We have previously demonstrated that mutation of G709 in TRPC6, which corresponds to G652 in TRPC3, generates a similar phenotype, featuring altered DAG sensitivity (Lichtenegger et al. 2018). The *new Figure EV3* shows an illustration, comparing the L2 site of TRPC3 and TRPC6 along with the sequence alignment of this region for all TRPC proteins. The glycine residue identified as critical for DAG recognition is indeed conserved throughout all TRPCs. Therefore, the conclusions of our current study are potentially relevant with respect to functional aspects for directly DAG-regulated (TRPC2/3/6/7) channels. This is now *addressed on page 7(end of Discussion)*.

(3) Figure 2 c, d: The high number of contacts of other lipids to residues in the L1 region (e.g. cholesterol) and in the L2 region (e.g. PC) are somewhat surprising. Is there any physiological relevance of these lipids in activation of TRPC3 channels?

We thank the reviewer for addressing this interesting aspect. Regulation of TRPC channels by DAG is suggested to involve reversible exchange of lipids within the coordination sites. Promiscuity of lipid coordination with respect to harboring lipid species is evident from the average numbers provided in Fig 2C/D. If DAG unbinds, another, most likely functionally inert, lipid is expected to occupy the space. This relationship is now addressed in the discussion (*1st paragraph, page 6*).

To our knowledge, there is so far no information available about the functional role of other structural lipids within the tetrameric TRPC3 complex. We expect that the identification of potentially regulatory lipids besides DAG, such as cholesterol, within L1 and L2 will stimulate further investigation of control of channel function by its lipid environment.

(4) Introduction last sentence on page 1: "Interestingly, DAG-induced activation has also been observed for TRPC4 and TRPC5, but only ...". Please add the correct reference here.

The correct reference (Storch et al. 2017) has been included (*page 2*).

The authors wish to thank the reviewer for her/his highly constructive and helpful comments.

Referee #2:

Members of the TRPC family of ion channels have been proposed to be directly activated by lipids including diacylglycerol (DAG), which is a lipid second messenger generated by phospholipase C activation. Recent single particle cryo-EM structures of TRPC3 revealed two lipid binding pockets, termed L1 and L2, located in distinct subregions of the transmembrane domain, however, whether and how these lipid coordination sites facilitate DAG-induced channel activation has remained elusive. In the current manuscript, Erkan-Candag et al use molecular dynamics (MD) simulations, structure-guided mutagenesis, a photo-switchable DAG analogue termed OptoDArG, and electrophysiology to probe the contribution of the L1 and L2 lipid coordination sites to DAG-mediated TRPC3 channel regulation. MD simulations with a coarse-grained TRPC3 model and varying percentages of DAG in the membrane revealed that DAG interacts with specific residues in the L1 and L2 sites. This information along with previously published data was used to guide mutagenesis of residues in the L1 and L2 sites aimed at reducing DAG binding, which were used for whole-cell patch clamp recordings. Erkan-Candag et al found that endogenously produced DAG via carbachol-indued muscarinic acetylcholine receptor activation yielded reduced activity for both L1 and L2 mutants compared to wild type (WT) with the L2 mutants being particularly striking. The authors interpreted this reduced activity as being due to reduced DAG binding at the L1 and L2 sites in these mutants. The authors then used OptoDArG, which can be activated with red light and inactivated with blue light, to have more precise, temporal control over the amount DAG available within the membrane. Patch clamping with OptoDArG showed that WT TRPC3 is sensitized after the first OptoDArG activation pulse, which was characterized by enhanced activation kinetics at the second OptoDArG activation pulse. Inconsistent with their endogenously produced DAG recordings, Erkan-Candag et al found that the L2 mutant G652A was more active than WT TRPC3 after the first pulse and more sensitized than WT TRPC3 after the second pulse, suggesting the G652A mutant is highly sensitized after exposure to DAG. This was confirmed using low intensity red light for the first pulse to activate a small fraction of OptoDArG insufficient to activate TRPC3 followed by a stronger second pulse to activate the channel. Sensitization of the G652A mutant was so strong that the authors had to pre-inactivate OptoDArG with a blue light pulse to abolish sensitization. From these collective studies with OptoDArG, the authors conclude that DAG binding to the L2 site sensitizes TRPC3 channels. Due to conflicting data with carbachol- or OptoDArG-mediated channel activity and lack of experimental data to determine how the L1 and L2 mutants affect DAG binding, the author's conclusions are not supported by their data and this manuscript is not suitable for publication at this time. I recommend reconsidering a revised manuscript after the following major points are addressed:

1. Inconsistent and contradictory results. In Figure 3, carbachol-mediated WT and mutant TRPC3 activity reveal reduced activity for L1 and L2 lipid site mutants, which was most pronounced for the L2 mutant G652A. However, Figures 4 and 5 show the G652A mutant has enhanced activity and sensitization than WT TRPC3 with the photo-switchable OptoDArG agonist. This disagreement needs to be addressed.

The authors thank the reviewer for addressing this aspect, and we would like to apologize for not having adequately outlined and discussed this complex issue. Mutation of G652 in TRPC3 was previously shown to alter the channels´ ability to recognize different DAG species (Lichtenegger et al 2018). The TRPC3 G652A mutant shows reduced sensitivity to endogenous DAG and SAG, while exhibiting enhanced sensitivity to other DAGs including DOG and also OptoDArG. Reduced sensitivity of G652A to endogenous DAG may not only originate from a reduced stability of DAG-L2 interactions, this could also come from enhanced interaction with other endogenous lipids at L2. Of note, the photochromic ligand OptoDArG indeed exhibits enhanced activity in the G652A mutant. Using OptoDArG as a photoswitchable probe, allowed us to precisely quantify the impact of the G652A mutation on activation

kinetics. This aspect is now addressed in detail in the results (*end of pages 3 and 4*) and in the discussion section (*end of page 6*).

If Figures 4 and 5 are correct, the authors have demonstrated that low levels of DAG do not sensitize WT TRPC3, and that they have built a high-affinity DAG sensor into TRPC3 with the G652A mutant. However, it appears the authors have misinterpreted this data to mean DAG sensitizes and has a high affinity for WT TRPC3 in the native L2 site, but it is difficult to evaluate their interpretations with the inconsistencies noted above.

As pointed out by the reviewer, we are indeed confident that the G652A mutation within L2 alters lipid recognition by TRPC3, resulting in enhanced “affinity“ for OptoDArG. Importantly, a sensitization process is evident for both TRPC3 wild type and mutant channels. From Figure 4C, we conclude that cis-OptoDArG, at levels below the threshold for channel opening (pre-pulse), is able to interact with the channels (wt as well as mutant) to induce a sensitized state that is available for rapid activation by subsequent exposure to higher levels of the activating DAG. The threshold for the sensitizing interaction with OptoDArG is reduced by the G652A mutation (*Figure 5A*). As G652 is a key residue in L2, we conclude that DAGs sensitize TRPC3 by interaction in L2. Hence, the mutation of G652 in TRPC3 alters the DAG recognition feature of TRPC3 and, in addition, affects channel sensitization at low DAG levels.

2. Unknown effect of L1 and L2 mutants on DAG binding. The authors assume the TRPC3 L1 and L2 mutants reduce DAG binding compared to WT channels, which they infer from the reduced activity in Figure 3. Since accurate interpretation of their results critically relies on knowing how the L1 and L2 mutants, especially G562A, affect DAG binding, the authors should perform the same MD simulations using their mutant channels.

As suggested by the reviewer, we extended our MD analysis to characterize the impact of the G652A mutation. The comparison between the simulations of wild type TRPC3 with the trajectories obtained for the G652A mutant provided clear evidence for an impact of the mutation on DAG-TRPC3 interaction. The results, now shown in Figure 3D, are in line with previous functional data suggesting G652 determines "DAG recognition" in terms of the preference of L2 to accommodate DAG molecules. Experimentally, the G652A mutation was found to alter the sequence of activity for different DAGs as channel activators (Lichtenegger et al 2018). Some types of DAG molecules, including SAG, showed reduced activity, while others including cis-OptoDArG show enhanced activity. Analysis of our MD data demonstrates that the wild type tetrameric complex has a higher affinity to SAG as it shows higher occupancy in L2. This DAG coordination is clearly reduced in the G652A mutant with a considerably lower chance to reach a triple occupancy level in the complexes (*results on page 4; Figure 3D*) and addressed in the discussion, (*page 7*). These data are in line with and confirm our finding of reduced PLC-mediated activation of G652A channels.

A DAG binding assay would greatly strengthen their data as well.

Experimentally, a meaningful DAG binding assay would require purification and reconstitution of functional tetramers. Of note, the L2 DAG binding region is only formed within correctly organized homotetramers, and DAG interaction will be highly dependent on the lipid environment created in the reconstitution. This approach is clearly beyond the scope of the current study.

3. Physiologically irrelevant DAG concentrations. PIP2 accounts for approximately 1% of the

lipids in the plasma membrane, however, the authors used 2% and 10% DAG in their MD simulations. The authors must include an explanation for why such high concentrations of DAG were used for their MD simulations.

We performed our MD simulations at DAG levels close to reported or expected levels. Based on lipidomics studies, DAG is expected to rise during signaling events to or even above 1% of the total membrane lipids (Sampaio et al. 2010), which is the global DAG level used in our simulations. Our MD simulations started with DAG at a level of 2% of the inner leaflet of the membrane where it was restricted to reside for the first 5 µs. Upon release of the positional restraint, DAG will be equally distributed between the leaflets resulting in a DAG level of 1% in terms of total membrane content. Similarly, PIP2 as a precursor for DAG is largely confined to the inner leaflet and amounts to about 2% of inner leaflet lipids. The simulations starting with 10% DAG in the inner leaflet correspond to 5% DAG of total membrane concentration. We used this higher concentration, because the high DAG concentration has the advantage of a faster diffusion-dependent kinetics, and allows us to identify binding sites of low affinity. We have now clearly stated that our MD simulations reflect a global concentration of 1% (and 5%) DAG within the membrane once the DAG is released from its positional restraint in the inner leaflet (*results pages 2 and 4, legends to Fig 1, 2 and 3*).

4. Unclear impact of L1 and L2 mutants on channel functionality. While the authors used TIRF microscopy to show L1 and L2 mutants localize to the plasma membrane, they did not confirm these mutants retain structural and functional integrity. This is particularly important for the L2 site, which is in the pore domain that undergoes critical gating-associated conformational changes. The authors should use GSK1702934A, a selective and commercially available agonist for TRPC3 that does not interfere with DAG activation, to validate channel activity in the mutants independent of DAG.

We agree with the reviewer that measurement of membrane currents is essential to verify functional integrity of the mutants. We tested all mutants for their ability to form functional channels by challenging the mutant channels with GSK1702934A. Of note, these current responses, by itself, did not reveal a specific impact of mutations on DAG regulation. Our test for DAG-independent activation of TRPC3 channels (*Appendix Figure S1B*) confirmed that all mutants, which were selected for further analysis in optical lipid clamp experiments, were able to generate GSK-induced membrane currents.

Minor points to be addressed: The authors refer to a P-value <0.05 as a significant difference rather than statistical significance. In Figure 1b, the authors do not say what the blue and green are representing in the figure legend. In Figure 2e, I394 is mislabeled as I384. Figure 3a needs clarification for how the data in the figure was generated for the box plots. It is available in the methods, but it would be useful to readers to have that information in the figure legend
.
These errors have been corrected in the new manuscript.

The authors wish to thank the reviewer for her/his highly constructive and helpful comments.

Referee #3:

This paper attempts to address a long-standing question on how diacylglycerols gate TRPC channels. The authors used TRPC3 as an example and applied molecular dynamics, site-directed mutagenesis, electrophysiological analysis with optical activation of a photoswitchable DAG analog they have previously developed to determine the involvement of the two putative lipid binding sites on TRPC3 based on the recently described cryo-EM structures. The work was performed beautifully, and the results complement well with the available high resolution TRPC

structures, which will add to our understanding of the gating mechanism of these important channels. The experimental results are interesting, but I have trouble understanding the implication of the mutations at the L2 site. 1) The effects of Y648F and G652A on currents induced by carbachol and OptoDArG are in conflict. While these mutations strongly reduced the carbachol-evoked TRPC3 current, they enhanced and accelerated the current induced by optical activation of OptoDArG. The reasons and implications of these results need to be discussed.

We thank the reviewer for his positive feedback and for this important comment. We have now more clearly outlined and discussed our concept, which is based on a previous study identifying G652 as a determinant of "DAG recognition" (Lichtenegger et al 2018). The G652A mutation was found to alter the sequence of agonist activity for different DAGs at TRPC3. Certain DAG molecules, including SAG showed reduced, while others including cis-OptoDArG show markedly enhanced activity (*now outlined on page 3*). Reduced SAG interactions within L2 as a consequence of the G652A mutation was now corroborated by our MD simulation approach. This is described in the results (*page 4, second paragraph*), illustrated in *new Figure 3D* and addressed in the discussion (*page 7*). We conclude that mutation of L2 (G652A) in TRPC3 alters the DAG recognition feature of TRPC3 and affects channel sensitization at low DAG levels.

2) If Y648F and G652A mutations are supposed to disrupt the binding of DAG to L2, why would they facilitate channel gating by the cis OptoDArG? Is cis OptoDArG supposed to bind better to the mutant than the wild-type channel?

As outlined above (answer to point 1) the G652A mutation in L2 modifies the DAG recognition profile, but not the general ability to accommodate DAG molecules. Our current functional characterization of L2 mutations corroborates their impact on the sequence of DAG agonist activities. L2 mutations were found to affect endogenous DAG (SAG) in a different manner as OptoDArG.

3) Page 4, "to affect the channel's lipidation" needs an apostrophe before "s".
This has been corrected.

4) Page 4, "This channel state persisted during further activating pulses (Suppl. Fig 2)". Is this supposed to be Suppl. Fig. 3? Also, "subsequent" may be a better word here than "further".
This has been corrected.

5) Page 5, "which displayed similarly accelerated current activation, but only minor potentiation of the maximum current amplitude". Which figure does this refer to?
This notion refers to Fig 4A (*now indicated on page 5*).

6) Page 5, "At higher DAG levels, the lipid mediator presumably increases the open probability of TRPC3 by an additional DAG-induced conformational change within the channel". It is not obvious to me how this works.

Since the channel is potentially able to accommodate 4 DAG molecules per tetramer within the L2 regions of the protomer interface, we speculate that a certain occupancy pattern and stoichiometry is required to induce functional states such as the sensitized state at low and the fully activated state at high DAG levels. This is now *discussed on page 7*.

7) Page 5, "presumably reflecting DAG induced opening of sensitized channels". How does this work? Does it still involve DAG binding to L2 or to a different site?

We cannot entirely exclude additional DAG interactions outside L2 (now stated in the discussion). Nonetheless, DAG binding to L2 sites in the four channel subunits is likely to result in DAG lipidation at different stoichiometries. It remains as yet unclear if all 4 sites of the tetrameric complex have to become filled for channel activation. We speculate that occupancy at a level lower than 4 DAG-per-TRPC3-channel could induce the observed sensitized state, which is available for transition to a fully activated (high Po) state by further DAG accommodation as *discussed on page 7, second paragraph*.

8) Page 5, "exposure is considered to lack any preexposure to cis-OptoDArG". This sentence is awkward and hard to understand.

The sentence was rephrased:
..”after illumination by blue light …., the bath solution lacked significant levels of cis OptoDArG”.

9) Page 6, "(Fan et al.)" needs the publication year.
This reference has been corrected.

10) Page 6, "to be clarified if full activation of TRPC3 involves...". Which condition is considered a "full activation of TRPC3"?

TRPC3 is known as a constitutively active channel, which displays a distinct, although very low open probability (Po) in the absence of PLC activity. Therefore, we speculate that the sensitized state displays a similarly low basal, constitutive activity, while a certain level of DAG lipidation induces “full activation” of TRPC3 at high Po. Basal and fully activated channel features have been characterized at the single channel level in Lichtenegger et al. 2018. This concept is now included in the Graphical Abstract.

11) Page 6 "striking contrast to the virtual photolipid-mediated channel activation process". What do you refer to as "the virtual photolipid-mediated channel activation process"? Is it shown in one of the figures or just an imaginary process?

The term “virtual” is indeed confusing and has been eliminated.

We thank the reviewer for her/his valuable comments.

1st Revision - Editorial Decision **5th Apr 2022**

Manuscript number: EMBOR-2021-54276V2
Title: Diacylglycerols sensitize TRPC3 channels by interaction with the L2 lipid coordination site
Author(s): Hazel Erkan-Candag, Amy Clarke, Oleksandra Tiapko, Mathias Gsell, Thomas Stockner, and Klaus Groschner

Dear Prof. Groschner

Thank you for your patience while we have reviewed your revised manuscript. As you will see from the reports below, the referees are now all positive about its publication in EMBO reports. I am therefore writing with an 'accept in principle' decision, which means that I will be happy to accept your manuscript for publication once a few minor issues/corrections have been addressed, as follows.

1) Your manuscript contains 5 figures and qualifies for publication in our Reports section. To make this possible, please combine the Results and Discussion section and keep our character limit of plus/minus 27,000 characters in mind (including spaces but excluding materials & methods and references).

2) Appendix
- Please provide page numbers, also in the table of content
- Please correct the nomenclature of the Appendix tables to 'Appendix Table S#'.
- Appendix Fig. S1: please add scale bars to panel A and define the size in the legend. In panel B you define a p-value in the legend that is not shown in the graph.

3) Please shorten the title to 100 characters incl. spaces and please describe your findings in the abstract in present tense.

4) You could reference the cryo-EM structure of TRPC3 as Data citation, i.e., add to the citation of the paper reporting the structure a data reference to the structure itself (see the paragraph on 'Data citation in https://www.embopress.org/page/journal/14693178/authorguide#referencesformat for more information).

5) Please add callouts to the panels of Fig. EV1 (A, B).

6) Please add the heading 'Expanded View Figure Legends'.

7) The synopsis image looks very good, but when I reduce the size to the final 550 pixels width, the label 'Ca2+' is not very well visible. Maybe changing the contrast or color could help.

8) Could you please also provide a draft for the summary text that accompanies your synopsis image online? We need a short (1-2 sentences) summary of the findings and their significance and 2-3 bullet points highlighting key results.

9) I attach to this email a related manuscript file with comments by our data editors. Please address all comments and upload a revised file with tracked changes with your final manuscript submission.

10) Please complete the information in the Author checklist, i.e., where in the manuscript the relevant information can be found.

Once you have made these minor revisions, please use the following link to submit your corrected manuscript:

Link Not Available

If all remaining corrections have been attended to, you will then receive an official decision letter from the journal accepting your manuscript for publication in the next available issue of EMBO reports. This letter will also include details of the further steps you need to take for the prompt inclusion of your manuscript in our next available issue.

Thank you for your contribution to EMBO reports.

Kind regards,

Martina Rembold, PhD
Senior Editor
EMBO reports

Referee #2:

The authors have addressed our concerns adequately. Largely, the issues seem to be due to not having clearly contextualized nor acknowledging their prior work as a conceptual launching point for this story.

Referee #3:

The authors have adequately addressed the concerns raised and the paper is acceptable for publication.

Dear Editors, dear Dr. Rembold,

thank you very much for your letter of April 5th with the 'accept in principle' decision on our manuscript. All minor open issues have been addressed and corrections were made as requested. Please find a detailed, account of our responses/corrections below. We hope that the corrected/modified version of our work is now suitable for publication in EMBO reports.
Thank you for your support and expert handling of our manuscript.

With kind regards on behalf of all authors

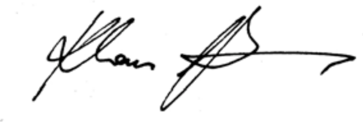

(Prof. Dr. Klaus Groschner)

List of responses/corrections

1) Your manuscript contains 5 figures and qualifies for publication in our Reports section. To make this possible, please combine the Results and Discussion section and keep our character limit of plus/minus 27,000 characters in mind (including spaces but excluding materials & methods and references).

Results and Discussion sections have been combined and an attempt was made to shorten the manuscript (currently 27,800 characters)

2) Appendix

- Please provide page numbers, also in the table of content
- Please correct the nomenclature of the Appendix tables to 'Appendix Table S#'.
- Appendix Fig. S1: please add scale bars to panel A and define the size in the legend. In panel B you define a p-value in the legend that is not shown in the graph.

All corrections have been introduced as requested.

3) Please shorten the title to 100 characters incl. spaces and please describe your findings in the abstract in present tense.

Title is now shortened <100 characters and own results are presented described in present tense (Abstract).

4) You could reference the cryo-EM structure of TRPC3 as Data citation, i.e., add to the citation of the paper reporting the structure a data reference to the structure itself (see the paragraph on 'Data citation in https://www.embopress.org/page/journal/14693178/authorguide#referencesformat for more information).

Reference has been added to the structure.

5) Please add callouts to the panels of Fig. EV1 (A, B).

Call-outs are now included.

6) Please add the heading 'Expanded View Figure Legends'.

The respective heading has been added

7) The synopsis image looks very good, but when I reduce the size to the final 550 pixels width, the label 'Ca2+' is not very well visible. Maybe changing the contrast or color could help.

We submit now a new, improved version of the synopsis image.

8) Could you please also provide a draft for the summary text that accompanies your synopsis image online? We need a short (1-2 sentences) summary of the findings and their significance and 2-3 bullet points highlighting key results.

Summary text and bullet points/key findings are now provided.

9) I attach to this email a related manuscript file with comments by our data editors. Please address all comments and upload a revised file with tracked changes with your final manuscript submission.

A new manuscript version with changes marked and answers to comments is submitted.

10) Please complete the information in the Author checklist, i.e., where in the manuscript the relevant information can be found.

Authors check list is completed.

2nd Revision - Editorial Decision 25th Apr 2022

Prof. Klaus Groschner
Medical University of Graz
Biophysics
Graz 8010
Austria

Dear Prof. Groschner,

I am very pleased to accept your manuscript for publication in the next available issue of EMBO reports. Thank you for your contribution to our journal.

At the end of this email I include important information about how to proceed. Please ensure that you take the time to read the information and complete and return the necessary forms to allow us to publish your manuscript as quickly as possible.

As part of the EMBO publication's Transparent Editorial Process, EMBO reports publishes online a Review Process File to accompany accepted manuscripts. As you are aware, this File will be published in conjunction with your paper and will include the referee reports, your point-by-point response and all pertinent correspondence relating to the manuscript.

If you do NOT want this File to be published, please inform the editorial office within 2 days, if you have not done so already, otherwise the File will be published by default [contact: emboreports@embo.org]. If you do opt out, the Review Process File link will point to the following statement: "No Review Process File is available with this article, as the authors have chosen not to make the review process public in this case."

Should you be planning a Press Release on your article, please get in contact with emboreports@wiley.com as early as possible, in order to coordinate publication and release dates.

Please also note that your paper might be eligible for open access publication in a way that is free of charge for the authors. Please contact either the administration at your institution or our publishers at Wiley (emboreports@wiley.com) for further questions.
(see also https://authorservices.wiley.com/author-resources/Journal-Authors/open-access/affiliation-policies-payments/institutional-funder-payments.html).

Thank you again for your contribution to EMBO reports and congratulations on a successful publication. Please consider us again in the future for your most exciting work.

Yours sincerely,

Martina Rembold, PhD
Senior Editor
EMBO reports

****

THINGS TO DO NOW:

Please note that you will be contacted by Wiley Author Services to complete licensing and payment information. The required 'Page Charges Authorization Form' is available here: https://www.embopress.org/pb-assets/embo-site/er_apc.pdf

You will receive proofs by e-mail approximately 2-3 weeks after all relevant files have been sent to our Production Office; you should return your corrections within 2 days of receiving the proofs.

Please inform us if there is likely to be any difficulty in reaching you at the above address at that time. Failure to meet our deadlines may result in a delay of publication, or publication without your corrections.

All further communications concerning your paper should quote reference number EMBOR-2021-54276V3 and be addressed to emboreports@wiley.com.

Should you be planning a Press Release on your article, please get in contact with emboreports@wiley.com as early as possible, in order to coordinate publication and release dates.

EMBO Press Author Checklist

Corresponding Author Name: Klaus Groschner	
Journal Submitted to: EMBO Reports	
Manuscript Number: EMBOR-2021-54276V2	

USEFUL LINKS FOR COMPLETING THIS FORM
The EMBO Journal - Author Guidelines
EMBO Reports - Author Guidelines
Molecular Systems Biology - Author Guidelines
EMBO Molecular Medicine - Author Guidelines

Reporting Checklist for Life Science Articles (updated January 2022)
This checklist is adapted from Materials Design Analysis Reporting (MDAR) Checklist for Authors. MDAR establishes a minimum set of requirements in transparent reporting in the life sciences (see Statement of Task: 10.31222/osf.io/9sm4x). Please follow the journal's guidelines in preparing your manuscript.
Please note that a copy of this checklist will be published alongside your article.

Abridged guidelines for figures
1. Data
The data shown in figures should satisfy the following conditions:
- the data were obtained and processed according to the field's best practice and are presented to reflect the results of the experiments in an accurate and unbiased manner.
- ideally, figure panels should include only measurements that are directly comparable to each other and obtained with the same assay.
- plots include clearly labeled error bars for independent experiments and sample sizes. Unless justified, error bars should not be shown for technical replicates.
- if n<5, the individual data points from each experiment should be plotted. Any statistical test employed should be justified.
- Source Data should be included to report the data underlying figures according to the guidelines set out in the authorship guidelines on Data Presentation.

2. Captions
Each figure caption should contain the following information, for each panel where they are relevant:
- a specification of the experimental system investigated (eg cell line, species name).
- the assay(s) and method(s) used to carry out the reported observations and measurements.
- an explicit mention of the biological and chemical entity(ies) that are being measured.
- an explicit mention of the biological and chemical entity(ies) that are altered/varied/perturbed in a controlled manner.
- the exact sample size (n) for each experimental group/condition, given as a number, not a range;
- a description of the sample collection allowing the reader to understand whether the samples represent technical or biological replicates (including how many animals, litters, cultures, etc.).
- a statement of how many times the experiment shown was independently replicated in the laboratory.
- definitions of statistical methods and measures:
  - common tests, such as t-test (please specify whether paired vs. unpaired), simple χ2 tests, Wilcoxon and Mann-Whitney tests, can be unambiguously identified by name only, but more complex techniques should be described in the methods section;
  - are tests one-sided or two-sided?
  - are there adjustments for multiple comparisons?
  - exact statistical test results, e.g., P values = x but not P values < x;
  - definition of 'center values' as median or average;
  - definition of error bars as s.d. or s.e.m.

Please complete ALL of the questions below.
Select "Not Applicable" only when the requested information is not relevant for your study.

Materials

Newly Created Materials	Information included in the manuscript?	In which section is the information available? (Reagents and Tools Table, Materials and Methods, Figures, Data Availability Section)
New materials and reagents need to be available; do any restrictions apply?	Not Applicable	

Antibodies	Information included in the manuscript?	In which section is the information available? (Reagents and Tools Table, Materials and Methods, Figures, Data Availability Section)
For **antibodies** provide the following information:<br>- Commercial antibodies: RRID (if possible) or supplier name, catalogue number and or/clone number<br>- Non-commercial: RRID or citation	Not Applicable	

DNA and RNA sequences	Information included in the manuscript?	In which section is the information available? (Reagents and Tools Table, Materials and Methods, Figures, Data Availability Section)
**Short novel DNA or RNA including primers, probes**: provide the sequences.	Not Applicable	

Cell materials	Information included in the manuscript?	In which section is the information available? (Reagents and Tools Table, Materials and Methods, Figures, Data Availability Section)
**Cell lines:** Provide species information, strain. Provide accession number in repository **OR** supplier name, catalog number, clone number, and/**OR** RRID.	Yes	Matherials am Methods
**Primary cultures:** Provide species, strain, sex of origin, genetic modification status.	Not Applicable	
Report if the cell lines were recently **authenticated** (e.g., by STR profiling) and tested for mycoplasma contamination.	Yes	Materials and Methods

Experimental animals	Information included in the manuscript?	In which section is the information available? (Reagents and Tools Table, Materials and Methods, Figures, Data Availability Section)
**Laboratory animals or Model organisms:** Provide species, strain, sex, age, genetic modification status. Provide accession number in repository **OR** supplier name, catalog number, clone number, **OR** RRID.	Not Applicable	
**Animal observed in or captured from the field:** Provide species, sex, and age where possible.	Not Applicable	
Please detail **housing and husbandry conditions**.	Not Applicable	

Plants and microbes	Information included in the manuscript?	In which section is the information available? (Reagents and Tools Table, Materials and Methods, Figures, Data Availability Section)
**Plants:** provide species and strain, ecotype and cultivar where relevant, unique accession number if available, and source (including location for collected wild specimens).	Not Applicable	
**Microbes:** provide species and strain, unique accession number if available, and source.	Not Applicable	

Human research participants	Information included in the manuscript?	In which section is the information available? (Reagents and Tools Table, Materials and Methods, Figures, Data Availability Section)
If collected and within the bounds of privacy constraints report on age, sex and gender or ethnicity for all study participants.	Not Applicable	

Core facilities	Information included in the manuscript?	In which section is the information available? (Reagents and Tools Table, Materials and Methods, Figures, Data Availability Section)
If your work benefited from core facilities, was their service mentioned in the acknowledgments section?	Not Applicable	

Design

Study protocol	Information included in the manuscript?	In which section is the information available? (Reagents and Tools Table, Materials and Methods, Figures, Data Availability Section)
If study protocol has been **pre-registered, provide DOI in the manuscript**. For clinical trials, provide the trial registration number **OR** cite DOI.	Not Applicable	
Report the **clinical trial registration number** (at ClinicalTrials.gov or equivalent), where applicable.	Not Applicable	

Laboratory protocol	Information included in the manuscript?	In which section is the information available? (Reagents and Tools Table, Materials and Methods, Figures, Data Availability Section)
Provide DOI OR other citation details if **external detailed step-by-step protocols** are available.	Not Applicable	

Experimental study design and statistics	Information included in the manuscript?	In which section is the information available? (Reagents and Tools Table, Materials and Methods, Figures, Data Availability Section)
Include a statement about **sample size** estimate even if no statistical methods were used.	Not Applicable	
Were any steps taken to minimize the effects of subjective bias when allocating animals/samples to treatment (e.g. **randomization procedure**)? If yes, have they been described?	Not Applicable	
Include a statement about **blinding** even if no blinding was done.	Not Applicable	
Describe **inclusion/exclusion criteria** if samples or animals were excluded from the analysis. Were the criteria pre-established?<br><br>If sample or data points were omitted from analysis, report if this was due to attrition or intentional exclusion and provide justification.	Not Applicable	
For every figure, are **statistical tests** justified as appropriate? Do the data meet the assumptions of the tests (e.g., normal distribution)? Describe any methods used to assess it. Is there an estimate of variation within each group of data? Is the variance similar between the groups that are being statistically compared?	Yes	Materials and Methods

Sample definition and in-laboratory replication	Information included in the manuscript?	In which section is the information available? (Reagents and Tools Table, Materials and Methods, Figures, Data Availability Section)
In the figure legends: state number of times the experiment was **replicated** in laboratory.	Yes	Figure legends
In the figure legends: define whether data describe **technical or biological replicates**.	Yes	Figure legends

Ethics

Ethics	Information included in the manuscript?	In which section is the information available? (Reagents and Tools Table, Materials and Methods, Figures, Data Availability Section)
Studies involving **human participants**: State details of **authority granting ethics approval** (IRB or equivalent committee(s), provide reference number for approval.	Not Applicable	
Studies involving **human participants**: Include a statement confirming that **informed consent** was obtained from all subjects and that the experiments conformed to the principles set out in the WMA Declaration of Helsinki and the Department of Health and Human Services Belmont Report.	Not Applicable	
Studies involving **human participants:** For publication of **patient photos**, include a statement confirming that consent to publish was obtained.	Not Applicable	
Studies involving experimental **animals**: State details of **authority granting ethics approva**l (IRB or equivalent committee(s), provide reference number for approval. Include a statement of compliance with ethical regulations.	Not Applicable	
Studies involving **specimen and field samples:** State if relevant **permits** obtained, provide details of authority approving study; if none were required, explain why.	Not Applicable	

Dual Use Research of Concern (DURC)	Information included in the manuscript?	In which section is the information available? (Reagents and Tools Table, Materials and Methods, Figures, Data Availability Section)
Could your study fall under dual use research restrictions? Please check biosecurity documents and list of **select agents and toxins** (CDC): https://www.selectagents.gov/sat/list.htm	Not Applicable	
If you used a select agent, is the security level of the lab appropriate and reported in the manuscript?	Not Applicable	
If a study is subject to dual use research of concern regulations, is the name of the **authority granting approval and reference number** for the regulatory approval provided in the manuscript?	Not Applicable	

Reporting

The MDAR framework recommends adoption of discipline-specific guidelines, established and endorsed through community initiatives. Journals have their own policy about requiring specific guidelines and recommendations to complement MDAR.

Adherence to community standards	Information included in the manuscript?	In which section is the information available? (Reagents and Tools Table, Materials and Methods, Figures, Data Availability Section)
State if relevant guidelines or checklists (e.g., **ICMJE, MIBBI, ARRIVE, PRISMA**) have been followed or provided.	Not Applicable	
For **tumor marker prognostic studies,** we recommend that you follow the **REMARK** reporting guidelines (see link list at top right). See author guidelines, under 'Reporting Guidelines'. Please confirm you have followed these guidelines.	Not Applicable	
For **phase II and III randomized controlled trials**, please refer to the **CONSORT** flow diagram (see link list at top right) and submit the CONSORT checklist (see link list at top right) with your submission. See author guidelines, under 'Reporting Guidelines'. Please confirm you have submitted this list.	Not Applicable	

Data Availability

Data availability	Information included in the manuscript?	In which section is the information available? (Reagents and Tools Table, Materials and Methods, Figures, Data Availability Section)
Have **primary datasets** been deposited according to the journal's guidelines (see 'Data Deposition' section) and the respective accession numbers provided in the Data Availability Section?	Not Applicable	
Were **human clinical and genomic datasets** deposited in a public access-controlled repository in accordance to ethical obligations to the patients and to the applicable consent agreement?	Not Applicable	
Are **computational models** that are central and integral to a study available without restrictions in a machine-readable form? Were the relevant accession numbers or links provided?	Yes	Materials and Methods
If publicly available data were reused, provide the respective **data citations in the reference list**.	Not Applicable	
